# The SDF-1α/MTDH axis inhibits ferroptosis and promotes the formation of anti-VEGF-resistant choroidal neovascularization by facilitating the nuclear translocation of SREBP1

**DOI:** 10.1007/s10565-025-10066-y

**Published:** 2025-07-17

**Authors:** Rong Zou, Xi Zhang, Xiaochan Dai, Yuanzhi Yuan, Jinhui Dai, Fei Yuan

**Affiliations:** 1 Department of Ophthalmology, Zhongshan Hospital, Fudan University, Shanghai, China; 2 Department of Ophthalmology, Shanghai Geriatric Medical Center, Shanghai, China; 3 Department of Ophthalmology, Zhongshan Hospital (Xiamen), Fudan University, Xiamen, China

**Keywords:** Age-related macular degeneration (AMD), Ferroptosis, Stromal cell-derived factor-1α (SDF-1α), Sterol regulatory element binding protein 1 (SREBP1), Metadherin (MTDH)

## Abstract

Age-related macular degeneration (AMD) has been well recognized as the first ranked blinding ocular fundus diseases among older individuals, particularly in developed regions, owing to its progressive nature and high prevalence in aging populations. Anti-vascular endothelial growth factor (VEGF) agents injected into patients’ vitreous cavity is the preferred treatment regimen for neovascular AMD. However, many patients exhibit resistance to anti-VEGF treatment, which is an urgent clinical problem. In this study, we treated mouse and endothelial cells with anti-VEGF drug Ranibizumab and stromal cell-derived factor-1α (SDF-1α) and found that ferroptosis was induced by Ranibizumab but inhibited by SDF-1α. SDF-1α inhibited ferroptosis by promoting transport of Sterol regulatory element binding protein 1 (SREBP1) from endoplasmic reticulum (ER) to Golgi transportation and SREBP1 maturation. Furthermore, we found that metadherin (MTDH) mediates SREBP1’ s movement from the endoplasmic reticulum (ER) to Golgi apparatus by inhibiting SREBP1 binding to INSIG1/INSIG2. Our study revealed the important role of SDF-1α/MTDH/SREBP1 axis in regulating anti-VEGF treatment resistance in patients with AMD.

**Graphical Abstract:**

1. SDF-1α mediates the development of anti-VEGF drug resistance by inhibiting ferroptosis.

2. SDF-1α reduces the sensitivity of endothelial cells to ferroptosis via the SREBP1/SCD1 signaling pathway.

3. SDF-1α promotes the maturation and nuclear translocation of SREBP1 through MTDH.

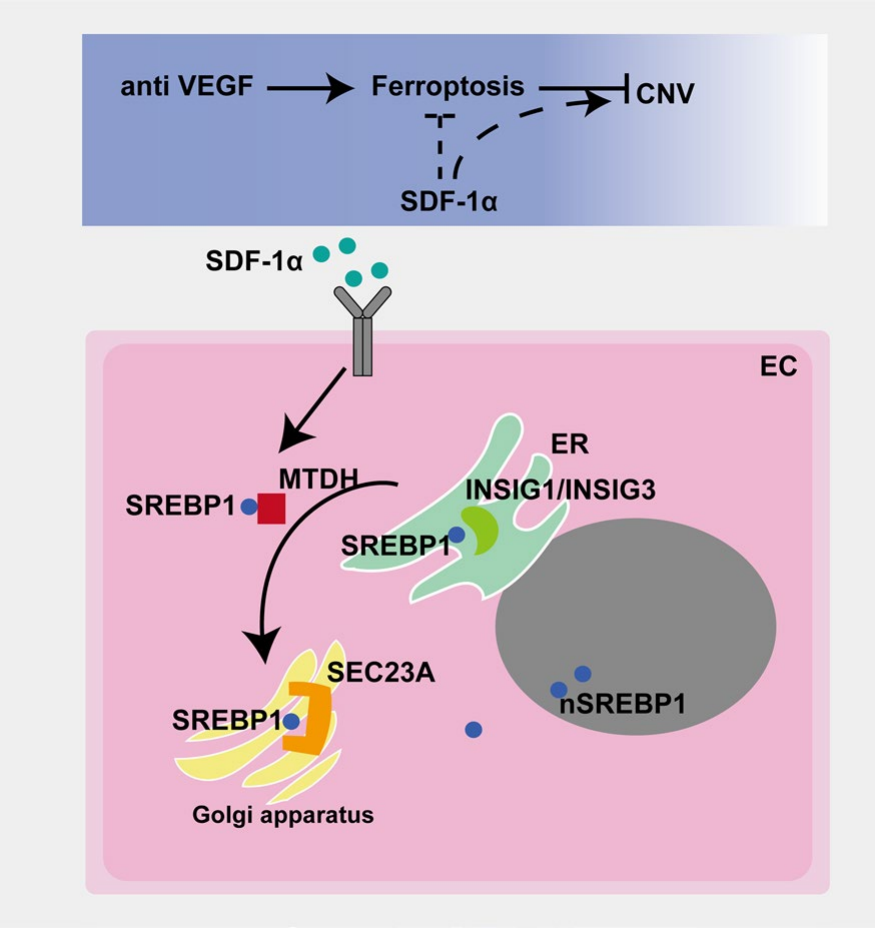

**Supplementary Information:**

The online version contains supplementary material available at 10.1007/s10565-025-10066-y.

## Introduction

As a prevalent degenerative ocular disorder in the aging adult population, age-related macular degeneration (AMD) is the principal contributor to severe visual impairment globally, and it is estimated that by 2040, there will be approximately 288 million patients with AMD worldwide (Fleckenstein et al. 2024). Neovascular AMD features dysregulated blood vessels in the macula at the late stage. Leakage from these immature vessels results in the accumulation of subretinal and intraretinal fluid, bleeding, and fibrosis, ultimately causing visual impairment (Flaxel et al. 2020). Intravitreal administration of anti-vascular endothelial growth factor (VEGF) compounds like Bevacizumab, Ranibizumab, and Aflibercept is the first-line treatment for neovascular AMD (mainly choroidal neovascularization (CNV)). However, 25% to 35% of patients display suboptimal response to anti-VEGF therapy (IRT) defined by the following criteria: (1) ongoing intraocular fluid effusion; (2) unresolved or de novo hemorrhage; (3) the lesion undergoing further fibrogenesis; or in combination with (4) the restoration of visual acuity failing to meet expectations (Mettu et al. 2021). To address this problem, it’s necessary to identifying the mechanisms of anti-VEGF treatment resistance in neovascular AMD.

The chemokine stromal Cell-Derived Factor-1 (SDF-1), alternatively named CXCL12, is a chemokine involved in multiple biological processes, encompassing cell migration, angiogenesis, and inflammation. Its role in AMD, particularly in neovascular (wet) AMD, has been increasingly studied (Yang et al. 2023). In laser-induced CNV mice, the ratio of endothelial progenitor cells and endothelial cells (ECs) was increased, and SDF-1 expression was upregulated, compared with control mice (Scotti et al. 2014). Based on our preliminary study and other team’s work, SDF-1 binds to its receptor CXCR4 or CXCR7 on endothelial cells, stimulating their migration and proliferation, which promotes pathological angiogenesis in choroidal neovascularization (CNV) (Li et al. 2021). SDF-1 can also increasing vascular permeability by weakening endothelial tight junctions (e.g., reducing claudin-5 and VE-cadherin expression) and ECs to secrete MMPs (e.g., MMP-2, MMP-9) in wet CNV (Chang et al. 2020). SDF-1-primed ECs upregulate adhesion molecules (e.g., VCAM-1, ICAM-1), enhancing infiltration of immune cells (e.g., macrophages, T cells) to the retinal choroid. These inflammatory cells release pro-angiogenic factors (e.g., TNF-α, IL-6), exacerbating ECs dysfunction and CNV growth (Zhong et al. 2013). SDF-1 signaling pathway is critical for CNV occurrence and development. However, whether SDF-1 is involved in anti-VEGF treatment resistance in CNV remains unexplored.

Ferroptosis represents a type of nonapoptotic cell death mechanism, distinguished by iron dependent membrane lipid peroxidation. Intracellular overload of free iron or iron-containing enzymes can result in iron reacting with oxygen and polyunsaturated fatty acids (PUFAs) lipids to drive excessive membrane lipid peroxides which accumulate and ultimately elicits cell death (Jiang et al. 2021). Because lipid peroxidation process is the mechanism by which ferroptosis leads to cell death, regulating lipid peroxidation is vital for protecting cells from ferroptosis (Jiang et al. 2021; Liao et al. 2022). Sterol regulatory element binding protein 1 (SREBP1) serves as a pivotal transcription factor for ferroptosis that regulates cholesterol biosynthesis, lipid homeostasis, and fatty acid synthesis (He et al. 2024). INSIG1/2, which localize to the endoplasmic reticulum (ER), bind the inactive SREBP1 precursor complex and disrupt the transport pathway from the endoplasmic reticulum (ER) to the Golgi apparatus (Xu et al. 2020). Conversely, SEC23A recognizes the SREBP1 complex and facilitates its transport to the Golgi apparatus (Lu et al. 2022). Furthermore, INSIG1/2 binding to SREBP1 suppresses the interaction between SREBP1 and SEC23A, thereby inhibiting SREBP1 transport (Sun et al. 2007). Thus, the interactions between of SREBP1 and INSIG1/2 or SEC23A are very important for SREBP1 transcription factor function. It has been reported that SREBP1/Stearoyl—CoA Desaturase 1 (SCD1) pathway reduces cellular sensitivity to ferroptosis by blocking lipid peroxidation and subsequent reactive oxygen species (ROS) accumulation (Chen et al. 2024).

Studies have found that, in the hypoxic microenvironment of CNV, mitochondrial fatty acid β-oxidation dysfunction in ECs promotes the buildup of PUFAs (such as arachidonic acid), which are easily oxidized by iron-dependent ROS into toxic lipid peroxides (Li et al. 2014; Liu et al. 2022). Additionally, downregulation of antioxidant enzymes (such as GPX4) makes ECs sensitive to ferroptosis (Zou et al. 2019). However, mild ferroptosis in the early stage of CNV stimulates ECs to secrete VEGF and PDGF, recruiting pericytes and immune cells to promote the formation of primitive vascular buds (Liu et al. 2024). Severe ferroptosis in the late stage of CNV causes ECs death, leading to abnormal neovascular structure (such as lack of pericyte coverage) and triggering leakage, hemorrhage, and fibrosis (Zheng et al. 2022). Recently, Hou, et.al conducted RNA sequencing of HepG2 cancer cells treated with Bevacizumab and found that Bevacizumab triggers cancer cells ferroptosis through the VEGF/PI3K/HAT1/SLC7A11 axis (Hou et al. 2024). Moreover, it has been proposed that, in tumors, reduced cellular sensitivity to ferroptosis has been correlated with resistance to cancer therapies (Zhang et al. 2022). Therefore, it is important to investigate whether SDF-1 mediates resistance to anti-VEGF drug therapy by inhibiting ferroptosis.

In the current study, we explored the mechanisms involving SDF-1α/SREBP1 that result in anti-VEGF treatment resistance by inhibiting ferroptosis in a mouse model of choroidal neovascularization (CNV) established by laser injury and in endothelial cells. This study elucidated the specific molecular mechanism through which SDF-1α suppresses ferroptosis to mediate anti-VEGF drug resistance, and provides a theoretical basis for combining anti-SDF-1α drugs with anti-VEGF drugs to treat AMD.

## Material and methods

### Animals and ethical approval

Eight-week-old male C57BL/6J mice were purchased from Shanghai Model Organisms Center, Inc. in Shanghai, China. The Shanghai Model Organisms Center, Inc. (SMOC) IACUC approved all animal experimental plans (July 20, 2024, Approved Number: 2024–0014). ARVO's Statement for the Use of Animals in Ophthalmic and Visual Research was strictly followed for every experimental methodology and animal husbandry protocol. Before conducting the experiments, the animals were allowed to adapt to the housing conditions spanning a duration of 7 days or more.

### Laser induced CNV model

Mice were anesthetized with 2% pentobarbital sodium solution administered intraperitoneally at 80 mg/kg (provided by SMOC, China), and the pupils were dilated using Compound Tropicamide Eye Drops. After fixing the eye in the appropriate position, a 5.4-mm handheld contact lens was placed over the cornea. A 532-nm frequency-doubled argon laser (parameters: power 100 mW, spot diameter 50 μm, exposure time of 100 ms) was then used to photocoagulation burns created circumferentially around the optic disc, 1–1.5 PD away from its margin. Following photocoagulation, a bubble (devoid of retinal hemorrhage) is formed, leading to disruption of the Bruch membrane, indicating effective establishment of a coagulated point.

### Vitreous delivery via injection

Under anesthesia, the mice were given eye antibiotic eye drops, and a 33-gauge syringe was vertically inserted to administer Anti-VEGF agent Ranibizumab (Novartis Pharma Schweiz AG) (1μL), Erastin (Selleck, USA, #S7242) (1μL) and SDF-1α (Biolegend, USA, # 589,806) (1uL) into the vitreous cavity via the equator of the eyeball.

### Retinal flat-mounting and immunofluorescence staining

Eyeball samples from mice were harvested and immersed in 4% Paraformaldehyde fixative for 3 h. With the help of an optical microscope, the retinas were carefully isolated. Then, for permeabilization and blocking, the retinas were subjected to overnight incubation at 4 °C in a solution containing 1% BSA and 0.5% Triton X-100. Next, the retinas were soaked in a solution containing FITC-labeled Lectin B4 (dilution ratio 1:100) (Vector labs, USA, # DL-1207) for an overnight duration at 4 degrees Celsius. PBS was utilized for three separate washing cycles and retinas were trimmed into a four-leaf clover shape. Then, the retinas were sealed with a fluorescent-protecting agent and stored at 4 ℃ in an environment free of ambient light. Finally, the retinas were observed, and photos were taken, using a laser confocal microscope.

### Tissue immunofluorescence staining

Histologically sections were taken of the eye and optic nerve. After dewaxing and antigen retrieval, a 5% BSA blocking buffer was applied to the tissues for 60 min. Thereafter, the tissues were probed with primary antibodies and incubated for 12 h (diluted as per the manufacturer’s recommended procedures) against the following proteins at 4 °C overnight: SREBP1(1:100, Invitrogen, USA, # PA1-337), GPX4 (1:100, Proteintech, China, #67,763–1-Ig), xCT (1:100, Proteintech, China, #26,864–1-AP), TFRC (1:100, Abcam, UK, #ab214039). Following three washes with PBS (5 min per wash), were then incubated with species-matched fluorescent secondary antibody. Then, the tissues were sealed with fluorescence-protecting agent and stored at 4 ℃ in an environment free of ambient light. The tissues were observed, and photos were taken, using a laser confocal microscope (Olympus FV3000).

### Cell and cell culture

The bEND.3 mouse brain microvascular endothelial cell line (BMECs), derived from murine brain tissue, was acquired from ATCC (CRL-2299). Cells between passage 11 and passage 30 are used for experiments.

**Ranibizumab treatment:** For subsequent experiments, BMECs were plated into 6-well plates at a seeding density of 3.6 × 10^5^ cells per well. In a standard culture setup, in a humidified 5% CO₂ incubator at 37 °C, BMECs were cultured in DMEM medium consisting of 10% FBS, 100 U/mL penicillin, and 100 μg/mL streptomycin for 24 h. On the second day, the growth medium was substituted with low-serum DMEM (2% FBS) for cell synchronization and Ranibizumab (1 μL/mL) for 24 h for treatment. For the blank control group, only complete medium (without Ranibizumab) is added. Other operations (such as cell density and culture conditions) are exactly the same as those in the Ranibizumab treatment group.

**Erastin, RSL3, SDF-1α treatment:** 3.6 × 10^5^ cells per well of BMECs were planted into a 6-well plate and 3.0 × 10^4^ cells per well of BMECs were planted into a 24-well plate. Cell culture conditions followed the earlier protocol in Ranibizumab treatment. On the second day, DMEM containing 2% FBS and Erastin (10 mM in DMSO) (Selleck, USA, #S7242), RSL3 (1 Mm in DMSO) (MedChemExpress, USA, # HY-100218A), or SDF-1α (100 μmol/L in distilled water) (Biolegend, USA, # 589,806) was used to renewed the culture medium, followed by a 24-h culturing period. For the groups treated with Erastin and RSL3, a volume of DMSO equal to that in the experimental group was pipetted into the control group’s medium. For the group treated with SDF-1α, the same volume of sterile distilled water was incorporated into the culture medium as part of the control group’s treatment protocol.

After 24 h, the cells were harvested, and RNA and protein isolation was performed for subsequent qRT PCR, immunoblotting detection or cellular immunofluorescence staining.

**siRNA transfection:** 3.6 × 10^5^ cells per well of BMECs were seeded into six-well plates. Then, 20 μM siRNA (siVEGFR2, siSREBP1, siMTDH) was added to OPTI-MEM Reduced Serum Medium (Gibco, USA) and mixed with Lipofectamine™ regent (ThermoFisher Scientific, USA, # L3000-008), and the mixture was added to the growth medium in the wells. After 48 h, proteins were collected and cell functions were assessed. The sequences of siRNA of VEGFR2, SREBP1 and MTDH used in the experiment are shown in Tables 1, 2 and 3:

**Table 1:** siRNA sequences targeting VEGFR2

	Sense Strand	Antisense Strand
SiVEGFR2-1	AAUUGUCAGUAUGUCUUUCUG	GAAAGACAUACUGACAAUUUU
SiVEGFR2-2	UGUUUUGCAGAAGAUACUGUC	CAGUAUCUUCUGCAAAACACU
SiVEGFR2-3	AGUGUUUUGCAGAAGAUACUG	GUAUCUUCUGCAAAACACUCA

**Table 2:** siRNA sequences targeting SREBP1

	Sense Strand	Antisense Strand
SiSREBP1-1	ACAAUCUUGUCAUUGAUAGAA	CUAUCAAUGACAAGAUUGUGG
SiSREBP1-2	AUUUAUUCAGCUUUGCUUCAG	GAAGCAAAGCUGAAUAAAUCU
SiSREBP1-3	UUUUGUGUGCACUUCGUAGGG	CUACGAAGUGCACACAAAAGC

**Table 3:** siRNA sequences targeting MTDH

	Sense Strand	Antisense Strand
SiMTDH-1	CCACCGAUGUUACAAGACA	UGUCUUGUAACAUCGGUGG
SiMTDH-2	CCCGAAGUAUAACUGCAAA	UUUGCAGUUAUACUUCGGG
SiMTDH-3	GGAUGUUAGCCGUAAUCAA	UUGAUUACGGCUAACAUCC

### RNA isolation and quantitative PCR

Cellular RNA was isolated using the RNA Extraction Kit (KeyGEN BioTECH, #KGR203) in strict accordance with the kit’s protocol. Subsequently, PCR amplification was carried out with TB Green® Premix Ex Taq™ (Takara Bio, Japan, #RR820A) on a fluorescence-based qPCR system. We programmed the thermal cycler with an initial 95 °C denaturation for 30 s, followed by 40 amplification cycles comprising 3 s at 95 °C and 30 s at 60 °C for annealing/extension. β-actin served as the housekeeping gene for normalization. To analyze the gene expression levels, we normalized the Ct values of each target gene to the corresponding β-actin Ct values from the same sample. Then, fold changes in gene expression were derived by calculating 2 ^− ΔΔCt^, where ΔΔCt = (CtTarget − CtReference) Treated − (CtTarget − CtReference) Control. The results were presented as relative fold changes, with the control group set as the baseline. All primer sequences used in this study are listed in Table 4.

**Table 4:** Primer sequences used in RT-qPCR

Gene		Sequence (5’−3’)
System xC	Forward	TGGTCCTAAATAGCACGAGT
	Reverse	TGTTTGCCCTTTAATTAGCTG
*FSP1*	Forward	ACCTTCCACAAATACTCAGGC
	Reverse	AGCTCCCTGGTCAGTAGCTC
*TFRC*	Forward	TGTGCAAACAATCTCAAGAGC
	Reverse	CTACAACATAACGGTCTGGT
*GPX4*	Forward	TGGCCTTCCCCTGCAACCAGT
	Reverse	CGCTTCACCACGCAGCCGTTC
*ACSL4*	Forward	GTTCATGATAAGCCGAACCC
	Reverse	AATTCTCCAATATCGCCAGT
*SREBP1*	Forward	CAAGGCCATCGACTACATCCG
	Reverse	TTTCATGCCCTCCATAGACACA
*SCD1*	Forward	ACGCCGACCCTCACAATT
	Reverse	CAGTTTTCCGCCCTTCTCTT
*β-actin*	Forward	CCAGGTCATCACTATTGGCAACGA
	Reverse	TCTTTACGGATGTCAACGTCACAC

### Immunoblotting

Proteins were collected from endothelial cells, and retina -choroid-sclera complex by Radioimmunoprecipitation Assay Lysis Buffer (RIPA) (KeyGEN BioTECH, Nanjing, China, #KGB5203-100) containing proteolytic enzyme inhibitors (Epizyme Biotech, Shanghai, China, #GRF101) and phosphatase-blocking agents (Epizyme Biotech, Shanghai, China, #GRF102). We calculated the loading volume for 40 µg of total protein using a BCA assay kit (KeyGEN BioTECH, Nanjing, China, # KGB2101-500). Protein samples were subjected to electrophoresis on a 12% SDS-PAGE gel (Epizyme Biotech, Shanghai, China, #PG113) using Tris–glycine running buffer (Epizyme Biotech, Shanghai, China, #PS105) at a constant voltage of 80 V for the first 30 min, then 120 V for the subsequent 60 min. Subsequently, proteins were transferred onto a PVDF membrane via wet transfer at a constant current of 200 mA for 2 h in the transfer buffer (Epizyme Biotech, Shanghai, China, #PS109). Then, the PVDF membrane (Millipore, Massachusetts, USA, #ISEQ00010) was immersed in a blocking solution containing 10% non-fat dried milk, reconstituted in Tris-buffered saline containing Tween 20 (TBST) (Epizyme Biotech, Shanghai, China, #PS103)) and gently shaken for 1 h at ambient temperature. Target-specific antibodies were judiciously selected in strict accordance with the defined experimental goals and pre-defined detection metrics stipulated in the research protocol (showed in Table 5). Working solution were used to incubate the PVDF membrane at 4 ℃ overnight. After washing the PVDF membrane with TBST solution, Horseradish peroxidase (HRP) labeled secondary antibodies incubate membrane at ambient temperature for a duration of 1 h. Washed the PVDF membrane with TBST solution again. Finally, the PVDF membrane was immersed in ECL reagent (Epizyme Biotech, Shanghai, China, #SQ201L) and incubated in the dark for 1 min. The chemiluminescence of protein bands in the PVDF membrane was observed using a Tanon 4600 series automatic imaging system for chemiluminescence and fluorescence analysis, and photographs were taken. Subsequently, Image J software was used to quantify the gray values.

**Table 5:** The antibodies used and their dilution ratios

Name	Supplier	dilution ratios
DyLight® 594 Griffonia Simplicifolia Lectin -Isolectin B4 (IB4)	Vector Lab	1:100
SREBP-1 antibody (A-4)	Santa Cruz	1:100
Anti-Ferritin antibody [EPR3004Y] (ab75973)	Abcam	1:100
Anti-Transferrin Receptor antibody [EPR20584] (ab214039)	Abcam	1:100
SLC7A11/xCT Polyclonal antibody	Proteintech	1:100
GPX4 Monoclonal antibody	Proteintech	1:100
INSIG1 Polyclonal antibody	Proteintech	1:100
INSIG2 Polyclonal antibody	Proteintech	1:100
Sec23A Rabbit mAb	ABclonal	1:50
Alexa Fluor-labeled goat anti-rabbit IgG (H + L) IgG(H + L)	Beyotime	1:100
Alexa Fluor-labeled goat anti-mouse IgG (H + L) IgG(H + L)	Beyotime	1:100

### Immunoprecipitation

The endothelial cells collected from the treatment and control groups in 1.5 mL centrifuge tubes underwent lysis through immunoprecipitation lysis buffer (Epizyme Biotech, Shanghai, China, #PC105) containing cocktail of inhibitors of protease (Epizyme Biotech, Shanghai, China, #GRF101) was added at a ratio of 30 μL per 1.0 × 10^5^ cells. After mixing, ice incubation of the samples was conducted for 20 min. Subsequently, the protein-containing lysate was collected by centrifugation at 12,000 × g for 10 min at 4 °C. The antibody (SREBP1 (Santa cruz, USA, #sc-365513), MTDH (Abcam, UK, #ab227981)) and protein A/G magnetic beads (Epizyme Biotech, Shanghai, China, #YG003) were sequentially added to the cell lysis buffer. The EP tubes were mounted on a rotator and subjected to incubation at room temperature for 2 h. Then clear, bead-free liquid was decanted and discarded and bead washing was carried out by immunoprecipitation lysis buffer. Cleaned magnetic beads were resuspend using immunoprecipitation lysis buffer containing protease inhibitors and the protein was diluted with loading buffer and then denatured by heating at 100 °C to facilitate subsequent Western blot detection.

### Cell proliferation assay

Cell viability was measured by CCK8 detection kit (KeyGEN BioTECH, Nanjing, China, # KGA9306-1000). Cells in each well of the 96 well plate were added with 10μL of CCK8 staining solution and placed in 37 ℃, 5% CO_2_ cell culture incubator for about 90 min. Lastly, we determined the OD value at 450 nm using the SpectraMax® iD3 continuous spectral multi-mode microplate detection platform from Molecular Devices. Calculate the proliferation activity of each group of cells using the formula: (Experimental well – Blank well)/(Control well – Blank well) × 100%.

### Cell migration assay using wound-healing method

When the cells had reached full confluency in six-well plates, straight lines were drawn in confluent cell monolayer along a ruler. After three washes using PBS, the cells were cultured in serum-reduced medium containing 1% FBS to minimize serum-induced effects. After 0, 1, 2, 3, 4, 6, 12, and 24 h, the cells were observed, and photos were taken, using an OLYMPUS BX63 microscope. Image J software was used to measure the scratch width and perform quantitative analysis.

### Matrigel tube formation assay

The Matrix-Gel™ Basement Membrane Matrix (Standard, Phenol Red-free) (Beyotime, Shanghai, China, # C0372) was removed from a −20 °C refrigerator to a 4 °C refrigerator until it thaws and melts into a liquid. Endothelial cells of control group and treated group were isolated and collected by 0.25% Trypsin–EDTA Solution (Phenol Red-free) (KeyGEN BioTECH, Nanjing, China, # KGL2101-100). Resuspend cells in complete culture medium and made sure cell density was 6 × 10^4^ cells/ml. Add 100 μL of Matrix-Gel™ Basement Membrane Matrix to the bottom of a 96-well plate, avoiding bubble formation. Then incubate the plate in a 37 ℃ incubator for 30 min to make the Matrix solid. 100 μL of cell resuspension was added to the surface of the Matrigel for 4 h’ incubation. Images were acquired using an OLYMPUS BX63 microscope. Image J software was utilized to determine the branches.

### ER and Golgi fluorescent probe incubation and cellular immunofluorescence staining

ER-Tracker Red probe (Beyotime, Shanghai, China, # C1041) and Golgi-Tracker Red probe (Beyotime, Shanghai, China, # C1043) were utilized in compliance with the kit’s instructions. First, we prepared the probe ready-to-use solution. Then, the medium was replaced with an aliquot of the working solution. After incubation, the cells were briefly fixed with a 4% paraformaldehyde solution. Then, the cells were blocked for 1 h, immersed in 5% BSA. Different antibodies targeting the target protein were added to cells (selected purposely chosen based on the pre-defined experimental goals and detection markers outlined in the research protocol) and placed at 4℃ overnight in the dark. After three PBS washes to remove unbound reagents, the cells were immersed in Alexa Fluor®-conjugated secondary antibodies for signal visualization. Then, the cells were sealed with a fluorescence-protecting agent and stored at 4 ℃ in dark. A laser confocal microscope (Olympus FV3000) was employed to detect images.

### Observation changes in mitochondrial morphology in endothelial cells using transmission electron microscopy (TEM)

Digest control and treatment group endothelial cells with 0.25% Trypsin–EDTA Solution (Phenol Red-free) to prepare single-cell suspensions. Collect cells by low-speed centrifugation of the cell suspension, and fix them with pre-cooled 2.5% glutaraldehyde for 2 h to stabilize mitochondrial structures within the cells. Next, rinse the cells 3 times with 0.1 M phosphate buffer for 15 min each to remove excess fixative. Post-fix the cells with 1% osmium tetroxide (OsO₄) for 1–2 h to enhance contrast. Subsequently, dehydrate the cells through a gradient of ethanol solutions (30%, 50%, 70%, 80%, 90%, 100%), with each concentration applied for 10–15 min. After dehydration, replace the ethanol with acetone twice, each for 10 min. Embed the cells in epoxy resin and polymerize them sequentially at 37 °C, 45 °C, and 60 °C. Use an ultramicrotome to cut ultra-thin Sects. (50–70 nm thick), mount the sections onto copper grids, and stain them with uranyl acetate and lead citrate for double staining. Finally, place the grids under a transmission electron microscope, focus stepwise from low to high magnification, observe the ultrastructural features of mitochondria (such as distribution, size, and cristae morphology within cells), and photograph the results for recording.

### Analysis of phosphorylation dynamics of proteins in endothelial cells by global phosphoproteomics

Collect cells with good viability, divide them evenly into a control group and an experimental treatment group, with 3 biological replicates for each group to reduce experimental errors and enhance the reliability of the results. After the treatment is completed, collect cell samples from each group separately. We discarded the culture medium and washed the cells three times with pre-cooled PBS. Then, cells were lysed on ice for 15–20 min with lysis buffer containing a protease inhibitor cocktail and phosphatase inhibitors. Meanwhile, perform ultrasonic disruption to further lyse the cells, fully release the intracellular proteins, and prevent changes in phosphorylation modifications. After lysis, centrifuge the samples at 12,000—15,000 rpm at 4 °C for 15—20 min. Collect the supernatant, measure the protein concentration using the BCA method, and adjust the protein concentrations of all samples to the same level.

Next, perform preliminary protein separation by SDS—PAGE electrophoresis. After electrophoresis, divide the gel lanes into several gel pieces according to the molecular weight range (e.g., each interval of 10—20 kDa) to ensure coverage of the entire proteome. Digest the gel pieces with trypsin to obtain a mixture of peptides. Employ TiO₂ (titanium dioxide) enrichment technology to specifically enrich phosphorylated peptides and remove interference from non—phosphorylated peptides. After desalting the enriched phosphorylated peptides, perform liquid chromatography—tandem mass spectrometry (LC—MS/MS) analysis. Process the mass spectrometry data using software such as MaxQuant and Proteome Discoverer. Identify phosphorylation sites by comparing with protein databases, and combine with TMT quantitative technology to analyze the differences in global proteome phosphorylation levels between the control group and the experimental treatment group.

### Reactive oxygen species (ROS) production analysis

One day prior to treatment, 50,000 cells were cultured into every well of 24-well culture dishes. Before treatment, cells were firstly immersed in diluted H2DCFDA (25 µM/500 µL Hanks Balanced Salt Solution (HBSS, Gibco®)) (Invitrogen™, USA, Cat. # C6827) at 37 °C for 30 min. Following it, the cells were subjected to corresponding drug or reagent treatments. Lastly, cellular observations and imaging were conducted using an OLYMPUS BX63 microscope.

### Assessment of lipid peroxidation by measuring malondialdehyde (MDA)

A lipid Peroxidation MDA Assay Kit (Beyotime, China, # S0131) was employed to determine the level of lipid peroxidation. One day prior to treatment, 200,000 cells were seeded into every well of six-well dishes. Upon completion of the 24-h treatment, the cells were collected using lysis buffer. The thiobarbituric acid (TBA) working solution was prepared, added to the cell lysis buffer, and mixed them. The mixture was heated at 100 °C for 15 min. Then, transfer 200 μL of the reagent into a 96-well plate and measure the OD value at 532 nm using a spectrophotometer.

### Statistical analysis

Data were derived from at least three independent experiments and are shown as the mean ± standard deviation (Mean ± SD). For quantitative data comparison between the control and treated groups, the independent samples t-test was employed. To assess data distribution, normality was verified via the Shapiro–Wilk test (*P* > 0.05), while Levene’s test (*P* > 0.05) was used to confirm homogeneity of variances. All results are presented as mean ± SD, and statistical analyses were performed using SigmaPlot 14.0 (Systat Software, San Jose, CA). A two-tailed P value < 0.05 was considered statistically significant.

## Results

### Ranibizumab inhibited laser-induced CNV but this effect was suppressed by SDF-1α.

**Fig. 1:**
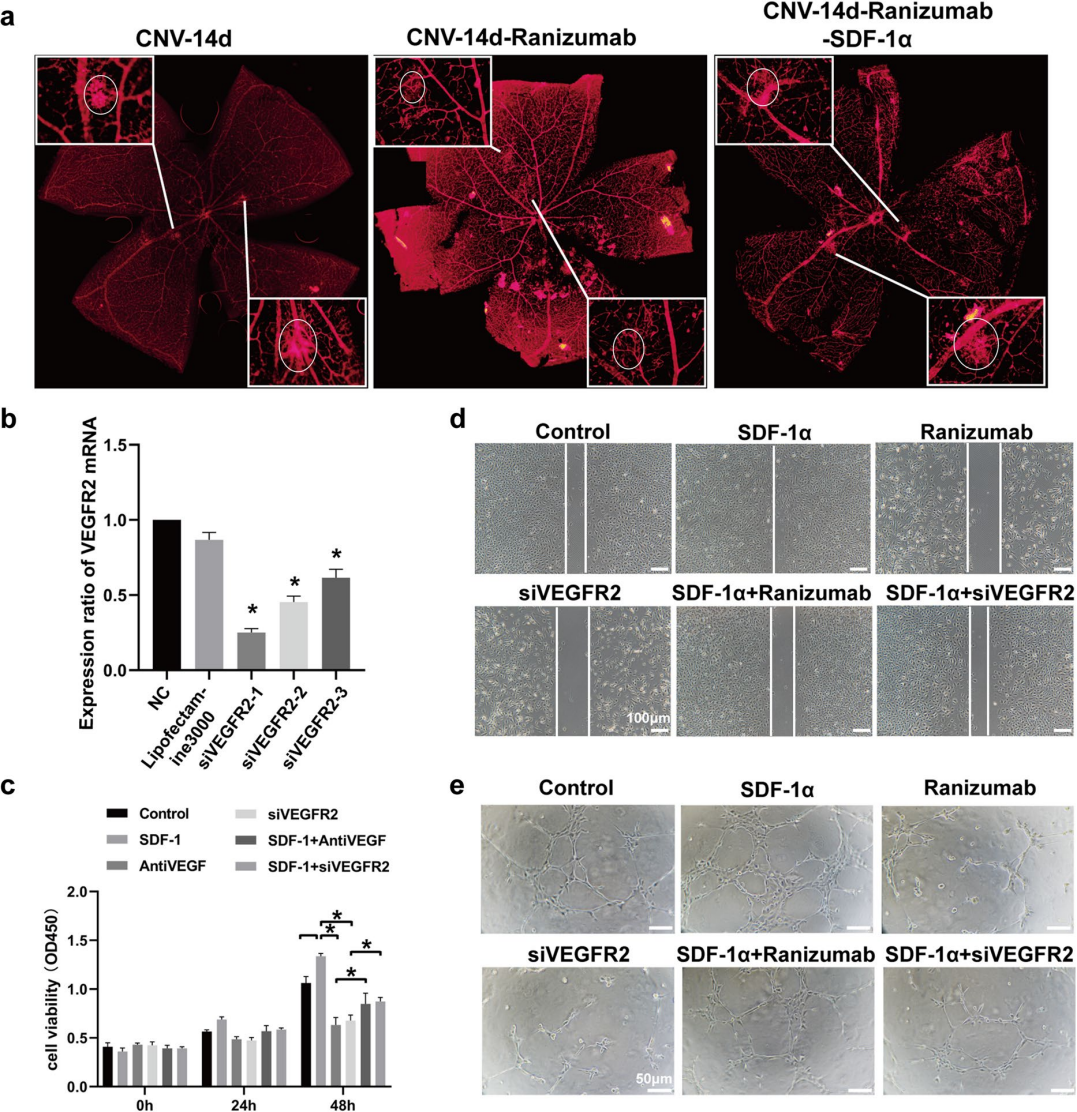
SDF-1α attenuates the anti-angiogenic effects of anti-VEGF antibody and VEGFR2 siRNA in vivo and in vitro. **a** A laser-induced choroidal neovascularization (CNV) mouse model was established. On day 1 after modeling, intravitreal injections of the anti-VEGF drug ranibizumab, and ranibizumab combined with SDF-1α were performed respectively. On day 14 after modeling, retinal whole-mount immunofluorescence staining (IB4: red) was used to visualize the size of CNV plaques (indicated by circles in the figures). **b** Three siRNAs targeting different loci of VEGFR2 (siVEGFR2-1、siVEGFR2-2、siVEGFR2-3) were constructed and transfected into endothelial cells. RT-PCR was performed to detect VEGFR2 mRNA levels and validate transfection and knockdown efficiency. **c** Endothelial cells were treated with distilled water, SDF-1α, the anti-VEGF drug ranibizumab, VEGFR2 siRNA, ranibizumab combined with SDF-1α, and VEGFR2 siRNA combined with SDF-1α. Cell proliferation activity was assessed by CCK8 assay through measuring the optical density (OD) at 450 nm at 24 and 48 hours after adding the CCK8 reagent. **d** Endothelial cells were treated as in (C), and cell migration ability was evaluated by scratch wound healing assay. The area of the scratch region was quantitatively analyzed using Image J software. **e** Endothelial cells were treated as in (C), and tube formation ability was assessed by matrigel tube formation assay. The number of tubes was quantitatively analyzed using Image J software. *p<0.05, **p<0.01, ***p<0.001

A laser-induced choroidal neovascularization mouse model was established in this study, and intravitreal injection of Ranibizumab or Ranibizumab with SDF-1α was performed on the second day. By day 14, the retinas were isolated, and retinal flat-mount immunofluorescence staining with IB4 was employed to reveal the dimension of macular CNV region. The results showed that the size of the macular CNV region in Ranibizumab injection group was notably smaller in size compared to the CNV group, and the size of CNV macular region in the Ranibizumab combined with SDF-1αgroup was significantly larger than the Ranibizumab group (Fig. 1A).

In vitro, we treated endothelial cells with SDF-1α, Ranibizumab, VEGFR2 siRNA, SDF-1α with Ranibizumab, or SDF-1α with VEGFR2 siRNA. CCK8 test, wound-healing assay and Matrigel tube-formation assay were performed to detect changes in endothelial cell viability, migration ability and tube-forming ability, respectively. The results showed that, in contrast to endothelial cells in the control group, the CCK8 test values of endothelial cells after SDF-1α treatment were much higher, while the values in the Ranibizumab and VEGFR2 siRNA treatment groups were much lower. However, compared with endothelial cells in the Ranibizumab and VEGFR2 siRNA treatment groups, both Ranibizumab and VEGFR2 siRNA combined with SDF-1α resulted in significantly higher CCK8 test values (Fig. 1B and C). The wound-healing test results showed that the distance between the cells on either side of the scratch was much narrower in the SDF-1α treatment group than in the control group, but the distance was much wider in Ranibizumab and VEGFR2 siRNA treatment group. Compared with endothelial cells in the Ranibizumab and VEGFR2 siRNA treatment groups, the scratch width in both the Ranibizumab group and the VEGFR2 siRNA with SDF-1α group was much narrower (Fig. 1D). The Matrigel tube formation assay showed that the number of tubes in SDF-1α treatment group was much greater than that in control group, while the number of tubes in Ranibizumab and VEGFR2 siRNA group were much lower. In contrasted with the endothelial cells in Ranibizumab and VEGFR2 siRNA treatment groups, the number of tubes in both Ranibizumab group and the VEGFR2 siRNA with SDF-1α group was significantly increased (Fig. 1E).

### SDF-1α inhibited ranibizumab-induced ferroptosis

**Fig. 2:**
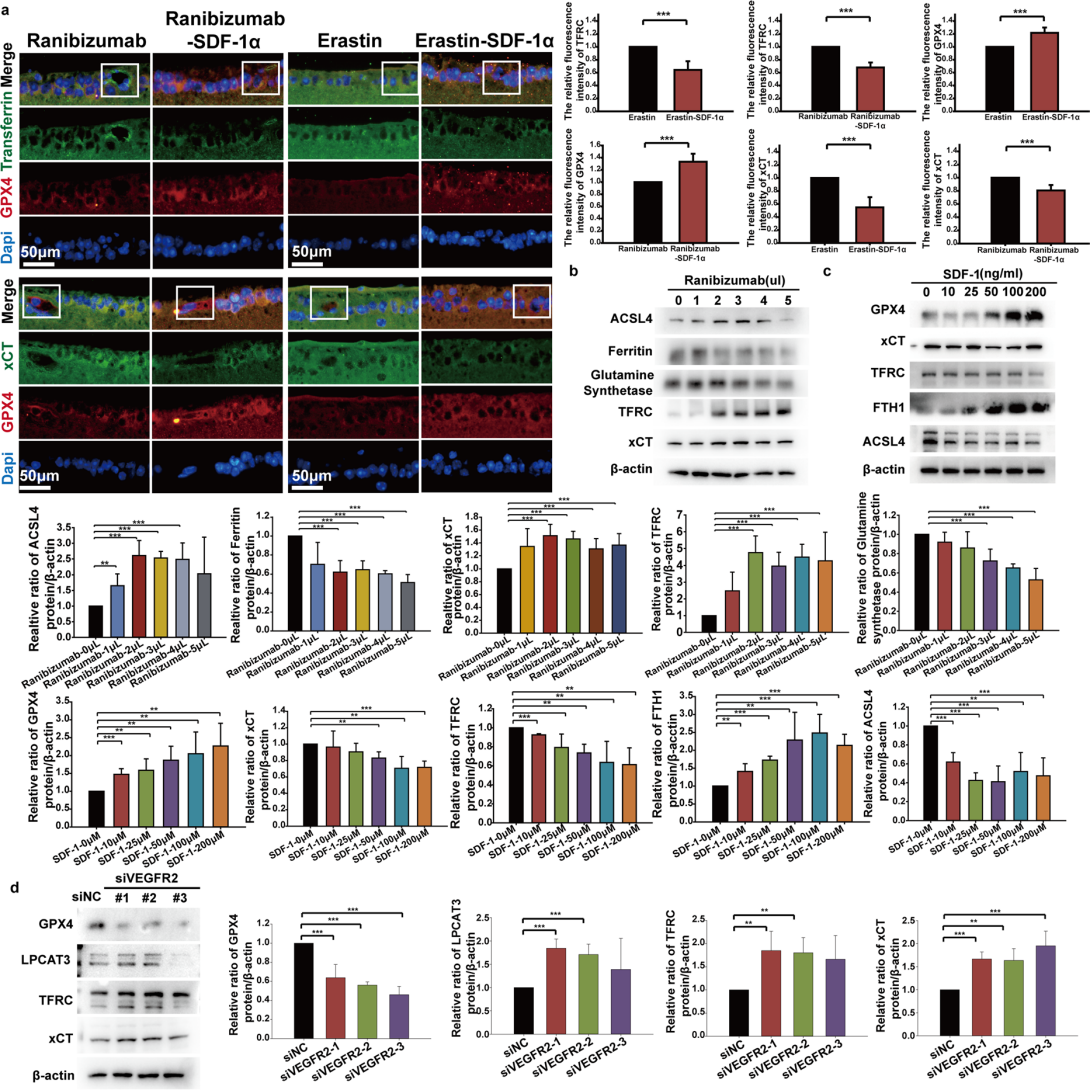
Opposing Regulatory Effects of Anti-VEGF treatment (Ranibizumab or VEGFR2 siRNA) and SDF-1α on Ferroptosis Molecular Markers. **a** Intravitreal injection of the anti-VEGF drug ranibizumab, ranibizumab combined with SDF-1α, the ferroptosis inducer Erastin, and Erastin combined with SDF-1α were administered into the eyes of mice (injected once every other day until day 7). On day 8, mouse eyes were enucleated, fixed, paraffin-embedded, and subjected to tissue sectioning for immunofluorescence staining (TFRC: green; GPX4: red; Dapi: blue). Fluorescence intensity in retinal tissues, particularly in retinal blood vessels, was observed and photographed using a fluorescence microscope (area within the box). Quantitative analysis of fluorescence intensity was performed using Image J software. **b** Endothelial cells were treated with 0, 1, 2, 3, 4, and 5 μL of ranibizumab for 24 hours. Cellular proteins were extracted, and the expression levels of ferroptosis molecular markers ACSL4, Ferritin, Glutamine synthetase, TFRC, and xCT were detected by immunoblotting. Quantitative analysis of band grayscale values was performed using Image J software. **c** Endothelial cells were treated with 0, 10, 25, 50, 100, and 200 ng/mL of SDF-1α for 24 hours. Cellular proteins were extracted, and the expression levels of ferroptosis molecular markers GPX4, ACSL4, FTH1, TFRC, and xCT were detected by immunoblotting. Quantitative analysis of band grayscale values was performed using Image J software. **d** siRNAs targeting three different sites of VEGFR2 (siVEGFR2-1, siVEGFR2-2, siVEGFR2-3) were constructed and transfected into endothelial cells for 48 hours. Cellular proteins were extracted, and the expression levels of ferroptosis molecular markers GPX4, LPCAT3, TFRC, and xCT were detected by immunoblotting. Quantitative analysis of band grayscale values was performed using Image J software. *p<0.05, **p<0.01, ***p<0.001

The anti-VEGF drug Ranibizumab, Ranibizumab combined with SDF-1α, Erastin, and Erastin combined with SDF-1α were injected into the vitreous of C57BL/6J mouse. Immunoblotting and immunofluorescence staining of retinal sections showed that Ferritin, TFRC and xCT were significantly upregulated and GPX4 was downregulated in Ranibizumab and Erastin groups versus control groups. These phenomena were reversed by SDF-1α (Fig. 2A).

**Fig. 3:**
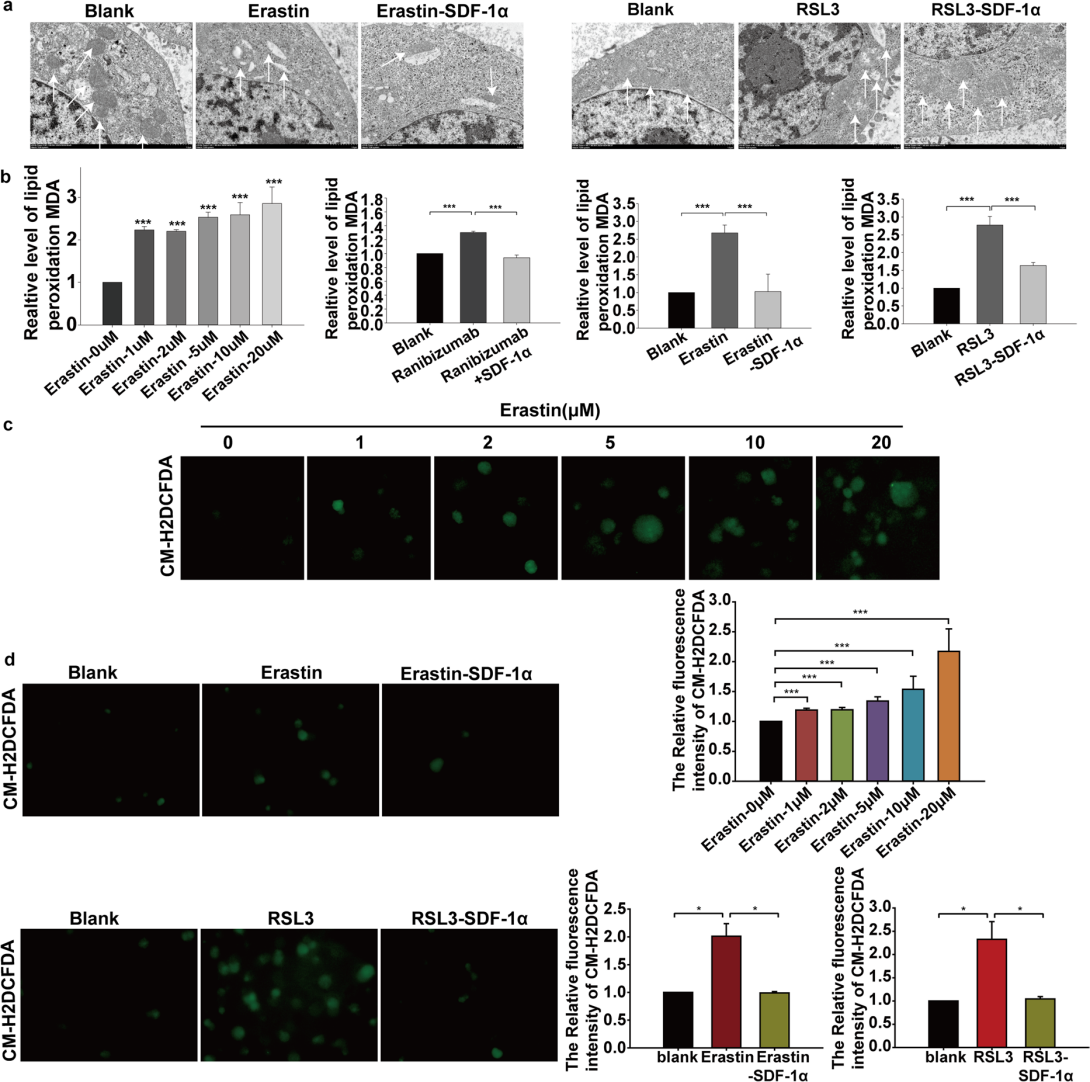
Role of SDF-1α in regulating lipid peroxidation metabolism induced by Erastin and RSL3. **a** Endothelial cells were treated with the ferroptosis inducers Erastin, RSL3, Erastin combined with SDF-1α, or RSL3 combined with SDF-1α for 24 hours, with DMSO-treated endothelial cells as the control group. After fixation, the morphology of mitochondria in endothelial cells was observed by transmission electron microscopy (TEM). **b** Endothelial cells were treated with 0, 1, 2, 5, 10, and 20 μM concentrations of Erastin, ranibizumab, ranibizumab combined with SDF-1α, Erastin, Erastin combined with SDF-1α, RSL3, or RSL3 combined with SDF-1α for 24 hours. The level of lipid peroxidation product malondialdehyde (MDA) in endothelial cells was detected using an MDA detection kit. **c**-**d** Endothelial cells were treated with 0, 1, 2, 5, 10, and 20 μM concentrations of Erastin, Erastin, Erastin combined with SDF-1α, RSL3, or RSL3 combined with SDF-1α for 24 hours. The production level of reactive oxygen species (ROS) in endothelial cells was detected using the ROS fluorescent probe CM-H2DCFDA. *p<0.05, **p<0.01, ***p<0.001

In vitro, we treated BMECs with 0 to 5 μL Ranibizumab, VEGFR2 siRNA and 0 to 200 ng/ml SDF-1α. Immunoblotting showed that molecules related to ferroptosis such as LPCAT3, TFRC and xCT were significantly upregulated in Ranibizumab and VEGFR2 siRNA groups, whereas other ferroptosis-related molecules such Ferritin, GPX4 and Glutamine Synthetase are downregulated (Fig. 2B and C). However, LPCAT3, TFRC and xCT were significantly downregulated by SDF-1α, and Ferritin, GPX4 and Glutamine Synthetase were upregulated, by SDF-1α (Fig. 2D). Then, endothelial cells were treated with Erastin and RSL3 to induce ferroptosis. PCR analysis showed that *TFRC* and *xCT* mRNA levels were decreased and *Ferritin、GPX4、Glutamine Synthetase* mRNA levels were increased. These phenomena were reversed by SDF-1α (Fig. 2E and F). Transmission electron microscope showed that both Erastin and RSL3 disrupted mitochondrial structure in endothelial cells, while SDF-1α reversed this effect (Fig. 3A). The level of lipid oxidation in endothelial cells was assessed by quantitative detection of Malondialdehyde (MDA), a product of membrane lipid peroxidation. The results showed that Erastin and RSL3 induced lipid peroxidation in endothelial cells; however, SDF-1α reduced the level of lipid peroxidation in these cells (Fig. 3B). CM-H2DCFDA is an ROS indicatorused to determine the cellular ROS production in this study. Both Erastin and RSL3 induced upregulation of ROS, and this effect was reversed by SDF-1α (Fig. 3C and D).

**Fig. 4:**
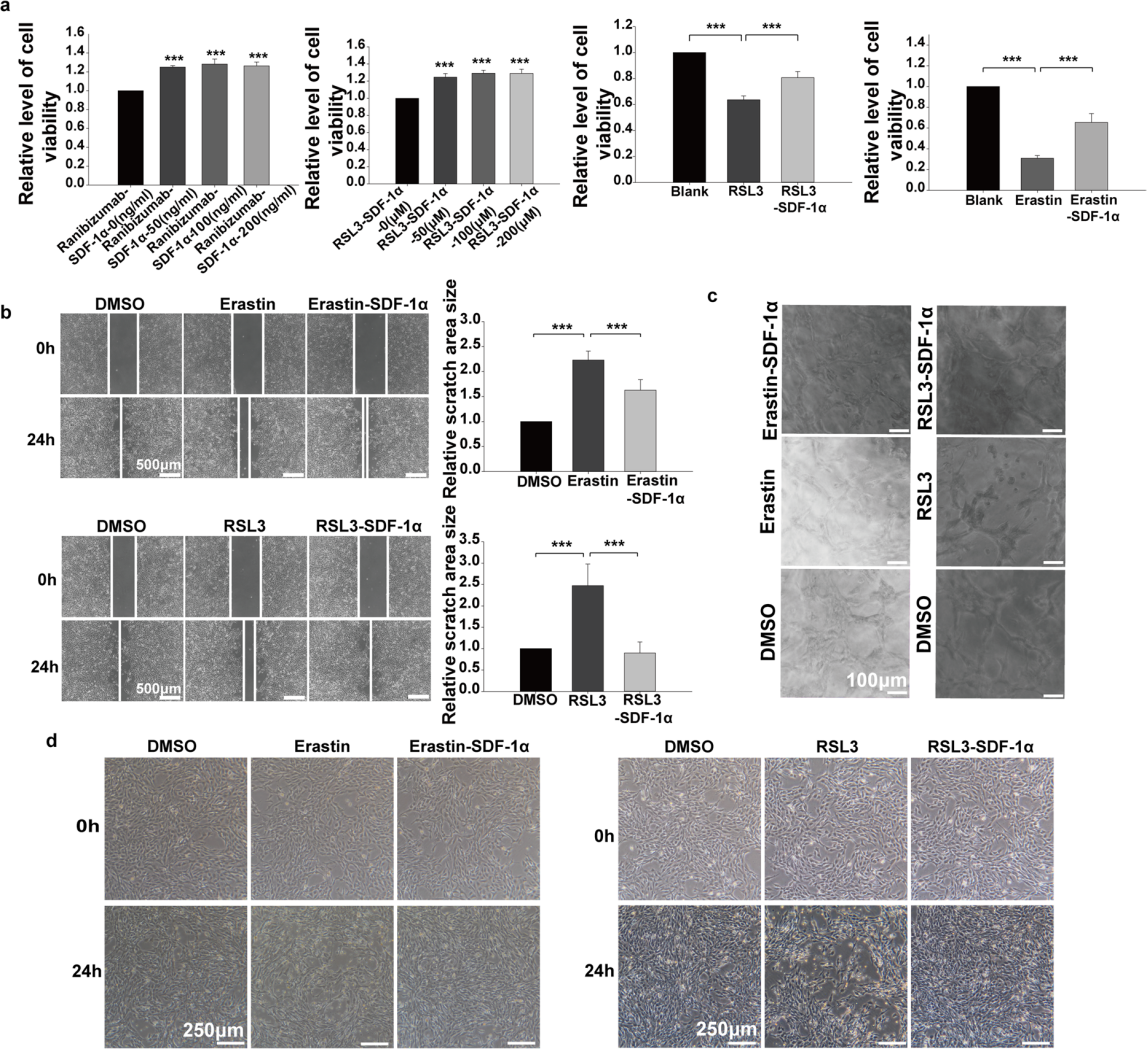
Role of SDF-1α on regulating viability, migration ability and tube-formation ability of endothelial cells changed by Erastin and RSL3. **a** Endothelial cells were treated with ranibizumab combined with SDF-1α (0, 50, 100, 200 μM), RSL3 combined with SDF-1α (0, 50, 100, 200 μM), Erastin, or Erastin combined with SDF-1α for 24 hours. The OD value at 450 nm was detected by CCK8 assay to explore the proliferation activity of endothelial cells. **b** Endothelial cells were treated with Erastin, Erastin combined with SDF-1α, RSL3, or RSL3 combined with SDF-1α for 24 hours. After scratching, the cells were continuously cultured, and images were taken at 0 hours and 24 hours after scratching. The area of the scratch region was quantitatively analyzed using Image J software. **c** Endothelial cells were treated with Erastin, Erastin combined with SDF-1α, RSL3, or RSL3 combined with SDF-1α for 24 hours. The tube formation ability of endothelial cells was detected by Matrigel tube formation assay, and the number of knots was quantitatively analyzed using Image J software. **d** Endothelial cells were treated with Erastin, Erastin combined with SDF-1α, RSL3, or RSL3 combined with SDF-1α for 24 hours. The growth status of endothelial cells was observed and photographed under an optical microscope. *p<0.05, **p<0.01, ***p<0.001

To determine the roles that SDF-1α plays in endothelial cells treated with anti-VEGF drugs and ferroptosis-inducing agents, CCK8 test, wound-healing assay, and Matrigel tube formation assay were performed. In contrasted to the endothelial cells in the control group, the CCK8 test values of endothelial cells after Erastin and RSL3 treatment were significantly lower. However, in contrast with the endothelial cells in the Erastin and RSL3 groups, both Erastin combined with SDF-1α and RSL3 combined with SDF-1α resulted in significant higher CCK8 test values (Fig. 4A). The wound-healing test results showed that the distance between the cells on either side of the scratch was much wider in the Erastin and RSL3 group in contrast to the blank group, while the distance was much narrower in the Erastin combined with SDF-1α group and the RSL3 combined with SDF-1α group compared with the Erastin and RSL3 group (Fig. 4B). Using the Matrigel tube formation assay, we observed fewer tubes in Erastin and RSL3 treatment group than that in the control group; however, the number of tubes in the Erastin combined with SDF-1α and RSL3 combined with SDF-1α groups were much greater than those in the Erastin and RSL3 group (Fig. 4C). Next, floating dead endothelial cells were observed using an optical microscope. Within comparison to the blank group, much more floating endothelial cells in the Erastin and RSL3 groups was shown. Significantly fewer floating endothelial cells were observed in the Erastin combined with SDF-1α group and the RSL3 combined with SDF-1α group than that in the Erastin and RSL3 groups (Fig. 4D).

### SDF-1α inhibited ferroptosis by promoting SREBP1 maturation and nuclear import

**Fig. 5:**
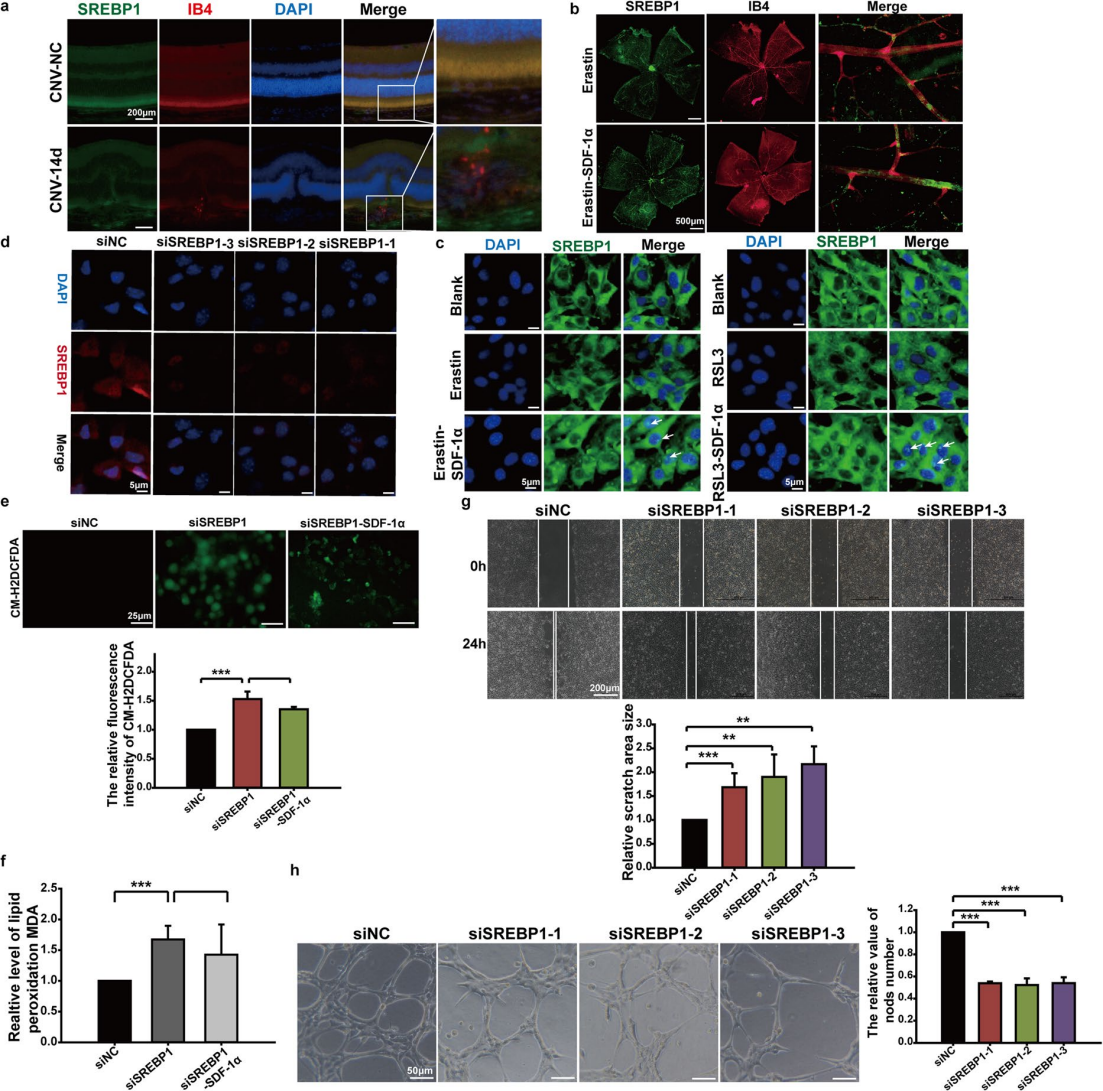
Effects of SREBP1 mediating SDF-1α regulating ferroptosis and endothelial cells functions. **a** A laser-induced CNV mouse model was established, and mouse eyes were enucleated and sectioned on day 14 after modeling. Immunofluorescence staining was performed to detect the expression level of SREBP1 in CNV plaques (SREBP1: green; IB4: red; Dapi: blue). **b** The ferroptosis inducer Erastin and Erastin combined with SDF-1α were intravitreally injected into mouse eyes (injected once every other day until day 7). On day 8, mouse eyes were enucleated, and retinal whole mounts were prepared followed by immunofluorescence staining (SREBP1: green; IB4: red). Fluorescence intensity in retinal blood vessels was observed and photographed using a fluorescence microscope. **c** Endothelial cells were treated with Erastin, Erastin combined with SDF-1α, RSL3, or RSL3 combined with SDF-1α for 24 hours. Cellular immunofluorescence staining was used to detect the fluorescence intensity and localization of SREBP1 in cells. **d** siRNAs targeting three different sites of SREBP1 (siSREBP1-1, siSREBP1-2, siSREBP1-3) were constructed and transfected into endothelial cells. The level of SREBP1 was detected by cellular immunofluorescence staining to validate transfection and knockdown efficiency. **e** After transfecting siSREBP1 into endothelial cells for 48 hours, cells were co-treated with SDF-1α for 24 hours. The production level of reactive oxygen species (ROS) in endothelial cells was detected using the ROS fluorescent probe CM-H2DCFDA, and quantitative analysis of fluorescence intensity was performed using Image J software. **f** After transfecting siSREBP1 into endothelial cells for 48 hours, cells were co-treated with SDF-1α for 24 hours. The level of lipid peroxidation product malondialdehyde (MDA) in endothelial cells was detected using an MDA detection kit. **g** siSREBP1 (siSREBP1-1, siSREBP1-2, siSREBP1-3) was transfected into endothelial cells for 48 hours, followed by scratching and continuous culture. Images were taken at 0 hours and 24 hours after scratching, and the area of the scratch region was quantitatively analyzed using Image J software. **h** siSREBP1 (siSREBP1-1, siSREBP1-2, siSREBP1-3) was transfected into endothelial cells for 72 hours. The tube formation ability of endothelial cells was detected by Matrigel tube formation assay, and the number of knots was quantitatively analyzed using Image J software. *p<0.05, **p<0.01, ***p<0.001

SREBP1, which is an important inhibitor of ferroptosis, was upregulated in neovascularization plaques on the 14th day after laser-induced choroidal neovascularization (Fig. 5A). In mice that received vitreous injection of Erastin or Erastin combined with SDF-1α, immunofluorescence staining for SREBP1 and the vascular dye Isolectin B4 (IB4) revealed significantly enhanced SREBP1 fluorescence intensity in retinal vasculature following combined Erastin/SDF-1α treatment compared to controls (Fig. 5B). Cellular immunofluorescence staining indicated that the nuclear fluorescence intensity of SREBP1 was greater in the endothelial cells from the Erastin/RSL3 combined with SDF-1α group compared with the Erastin/RSL3 group (Fig. 5C). Next, SREBP1 in endothelial cells were inhibited by siRNA transfection (Fig. 5D). CM-H2DCFDA analysis indicated that, after transfection, the ROS level in transfected cells was upregulated in contrasted to the negative control group, which was induced by SDF-1α (Fig. 5E). Quantitative detection of Malondialdehyde (MDA) showed that lipid peroxidation of endothelial cells was increased in transfected cells, however SDF-1α reduced the level of lipid peroxidation in these cells (Fig. 5F). Moreover, wound-healing test and Matrigel tube formation assays results demonstrated significantly impaired migration in SREBP1-silenced endothelial cells compared to negative controls; endothelial cells transfected with SREBP1 siRNA exhibited a significantly reduced number of tubes compared to the negative control group (Fig. 5G and H).

**Fig. 6:**
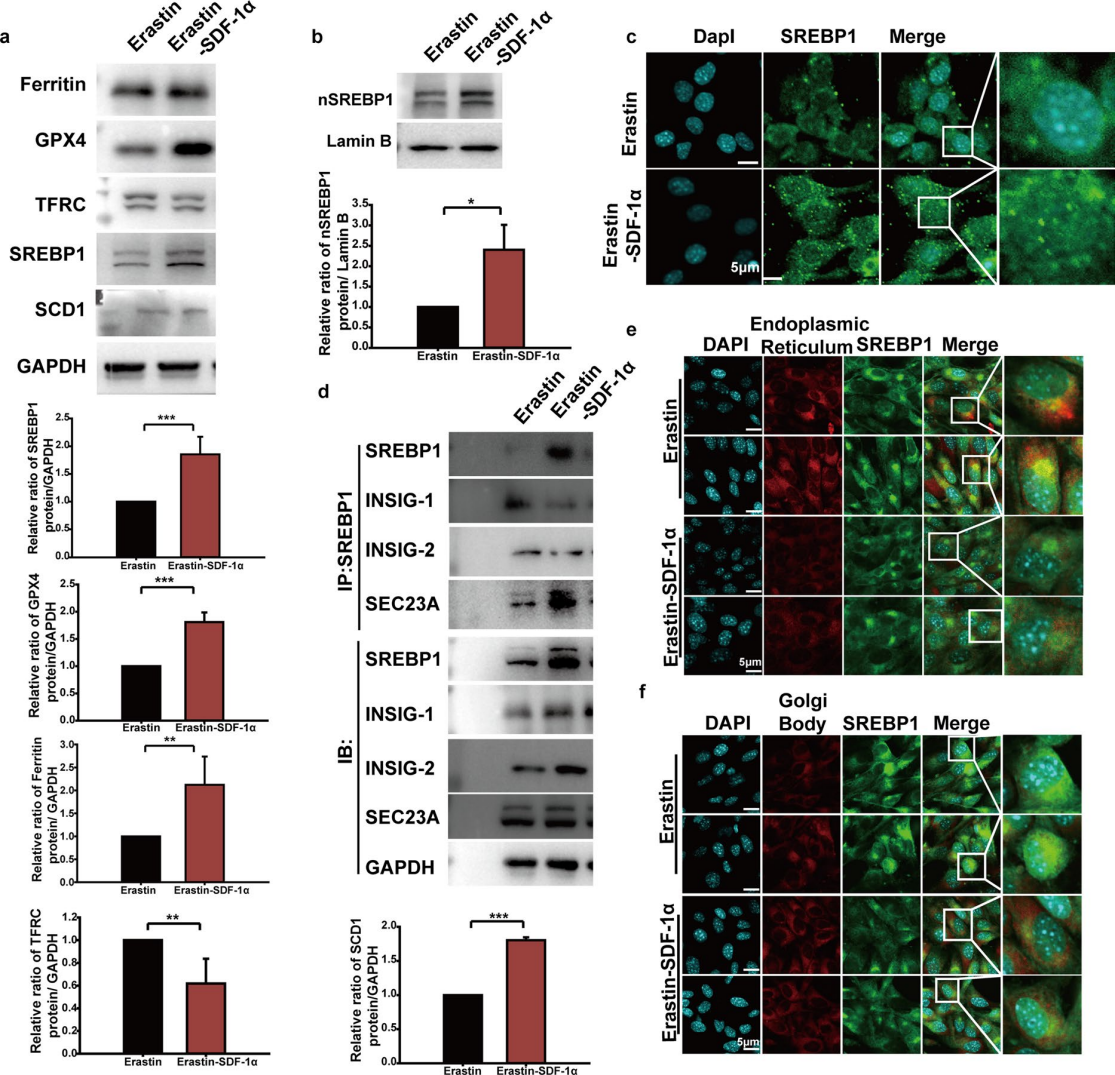
Effects of SDF-1α on ferroptosis and mature of SREBP1. **a** Endothelial cells were treated with Erastin or Erastin combined with SDF-1α for 24 hours. Cellular proteins were extracted, and the expression levels of ferroptosis markers (GPX4, Ferritin, TFRC, xCT) and molecules (SREBP1, SCD1) were detected by immunoblotting. Quantitative analysis of band grayscale values was performed using Image J software. **b** Endothelial cells were treated with Erastin or Erastin combined with SDF-1α for 24 hours. Nuclear proteins were extracted, and the level of SREBP1 in the nucleus was detected by immunoblotting. Quantitative analysis of band grayscale values was performed using Image J software. **c** Endothelial cells were treated with Erastin or Erastin combined with SDF-1α for 24 hours. Cellular immunofluorescence staining was used to detect the fluorescence intensity and localization of SREBP1 in cells. **d** Endothelial cells were treated with Erastin or Erastin combined with SDF-1α for 24 hours. Cellular proteins were extracted, and the binding of SREBP1 to its transport-related key molecules (INSIG1, INSIG2, and SEC23A) was identified by co-immunoprecipitation (Co-IP) followed by immunoblotting. **e** Endothelial cells were treated with Erastin or Erastin combined with SDF-1α for 24 hours. The subcellular localization of SREBP1 was detected by co-immunofluorescence staining using endoplasmic reticulum (ER) probes. **f** Endothelial cells were treated with Erastin or Erastin combined with SDF-1α for 24 hours. The subcellular localization of SREBP1 was detected by co-immunofluorescence staining using Golgi fluorescent probes

We treated endothelial cells with Eastin combined with SDF-1α. Immunoblotting showed that Ferritin, GPX4, SREBP1 and SCD1 were upregulated, while TFRC was downregulated, by SDF-1α (Fig. 6A). Immunoblotting for nuclear proteins indicated that nuclear SREBP1 was upregulated by SDF-1α (Fig. 6B). Cellular immunofluorescence staining demonstrated that nuclear SREBP1 fluorescence intensity was increased by SDF-1α (Fig. 6C). The SREBP1 immunoprecipitation and immunoblotting experiments showed that SREBP1 bound less INSIG1 and INSIG2, but more SEC23A, in the Erastin + SDF-1α group versus the Erastin group (Fig. 6D). Because ER-to-Golgi transportation is important for SREBP1 maturation and nuclear import, SREBP1 cellular localization was assessed using immunofluorescence staining with either ER-Tracker Red (endoplasmic reticulum marker) or a Golgi apparatus fluorescent probe (ER-Tracker Red) or a Golgi body fluorescence probe (Golgi-Tracker Red). The results showed that SREBP1 localization to the endoplasmic reticulum was decreased (Fig. 6E), while the SREBP1 localization to the Golgi body was enhanced, in the Erastin + SDF-1α group versus the Erastin group (Fig. 6F).

### SDF-1α promoted MTDH binding to SREBP1 and inhibitd ferroptosis

**Fig. 7:**
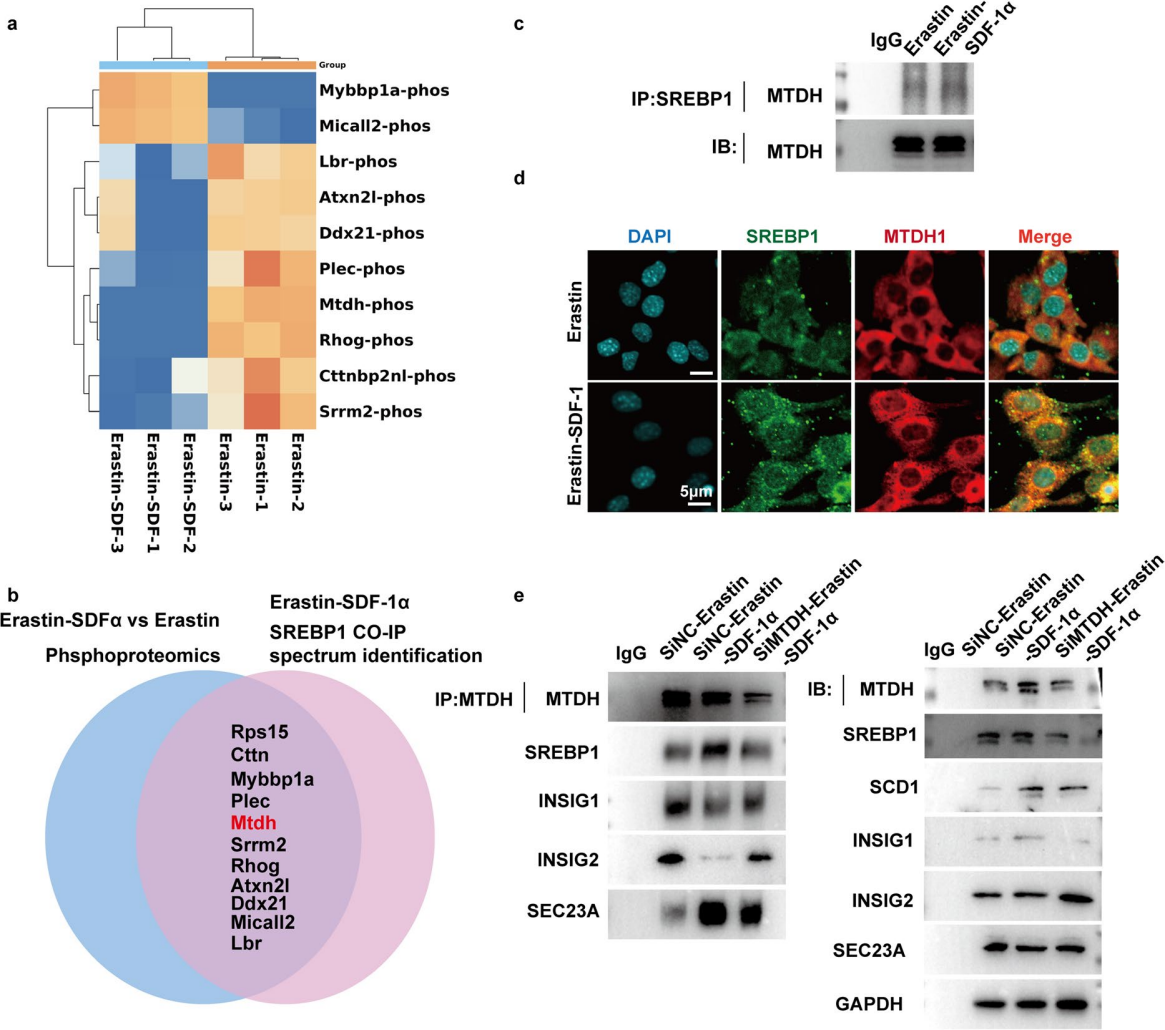
Effects of MTDH on SREBP1 combining with INSIG1, INSIG2 and SEC23A under SDF-1α and Erastin treatment. **a** Endothelial cells were treated with Erastin or Erastin combined with SDF-1α for 24 hours. Cellular proteins were extracted, and changes in the expression levels of phosphorylation-related molecules in endothelial cells were detected by phosphoproteomics. **b** Endothelial cells were treated with Erastin or Erastin combined with SDF-1α for 24 hours. Cellular proteins were extracted, and proteins bound to SREBP1 were identified by co-immunoprecipitation (Co-IP) combined with mass spectrometry. **c** Endothelial cells were treated with Erastin or Erastin combined with SDF-1α for 24 hours. Cellular proteins were extracted, and the binding of SREBP1 to MTDH in cells was validated by co-immunoprecipitation (Co-IP) followed by immunoblotting. **d** Endothelial cells were treated with Erastin or Erastin combined with SDF-1α for 24 hours. Cellular immunofluorescence staining was used to detect the fluorescence intensity and co-localization of SREBP1 and MTDH in cells. **e** Endothelial cells were treated with Erastin or Erastin combined with SDF-1α for 24 hours. Cellular proteins were extracted, and the binding of SREBP1 to its transport-related key molecules (INSIG1, INSIG2, and SEC23A) was validated by co-immunoprecipitation (Co-IP) followed by immunoblotting

To identify the mechanism by which SDF-1α inhibited SREBP1 binding toINSIG1/INSIG2 or SEC23A, total proteins was isolated from endothelial cells treated with Eastin combined with SDF-1α or Erastin alone, and phosphoproteomics analysis was performed. The proteins that immunoprecipitated with anti-SREBP1 antibody in the group treated with Erastin combined with SDF-1α or the group treated with Erastin alone were analyzed by spectrum technology. The results showed that MTDH phosphorylation was inhibited and MTDH binding to SREBP1 was enhanced by SDF-1α (Fig. 6A and B). A previous study showed that MTDH localization to the endoplasmic reticulum promotes cell proliferation and migration (Ahmed et al. 2024). Furthermore, the SREBP1 immunoprecipitation and immunoblotting showed that SREBP1 bound more MTDH in the Erastin + SDF-1α group versus the Erastin group (Fig. 7C). In vivo, immunofluorescence staining showed that co-localization of SREBP1 and MTDH was enhanced in the Erastin + SDF-1α group versus the Erastin group (Fig. 7D). The SREBP1 immunoprecipitation and immunoblotting experiments showed that SREBP1 bound more INSIG1/INSIG2 and less SEC23A in MTDH siRNA–transfected cells treated with Erastin and SDF-1α compared with the negative control cells (Fig. 7E).

**Fig. 8:**
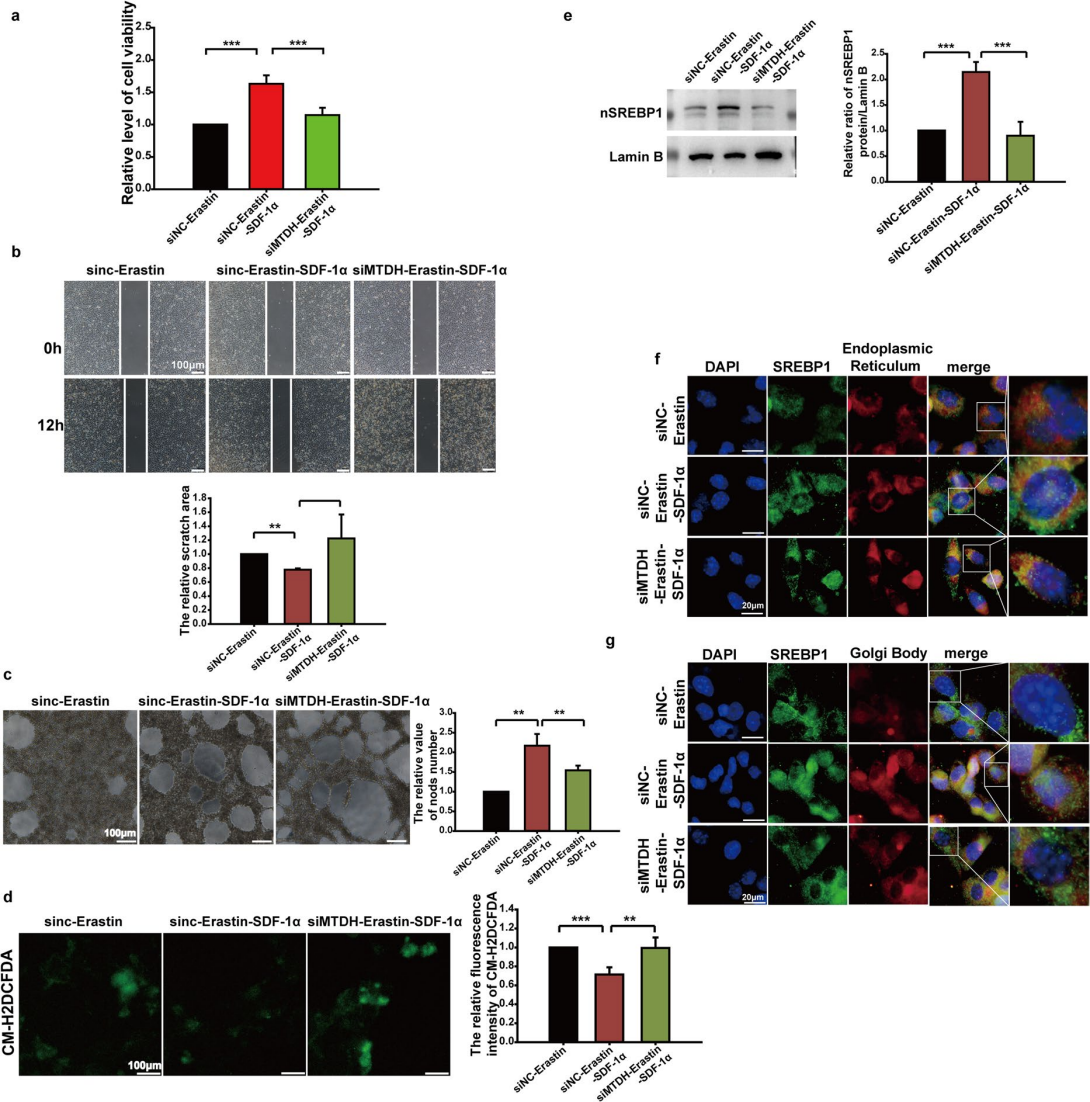
Effects of MTDH on SDF-1α inducing SREBP1 mature, ferroptosis and endothelial cells functions. **a** Endothelial cells were treated with Erastin, Erastin combined with SDF-1α, or Erastin combined with SDF-1α after transfection with MTDH siRNA. The OD value at 450 nm was detected by CCK8 assay to evaluate the proliferation activity of endothelial cells. **b** Endothelial cells were treated with Erastin, Erastin combined with SDF-1α, or Erastin combined with SDF-1α after transfection with MTDH siRNA. After scratching, cells were continuously cultured, and images were taken at 0 hours and 24 hours post-scratching. The area of the scratch region was quantitatively analyzed using Image J software. **c** Endothelial cells were treated with Erastin, Erastin combined with SDF-1α, or Erastin combined with SDF-1α after transfection with MTDH siRNA. The tube formation ability of endothelial cells was assessed by Matrigel tube formation assay, and the number of knots was quantitatively analyzed using Image J software. **d** Endothelial cells were treated with Erastin, Erastin combined with SDF-1α, or Erastin combined with SDF-1α after transfection with MTDH siRNA. The production level of reactive oxygen species (ROS) in endothelial cells was detected using the ROS fluorescent probe CM-H2DCFDA, and fluorescence intensity was quantitatively analyzed using Image J software. **e** Endothelial cells were treated with Erastin, Erastin combined with SDF-1α, or Erastin combined with SDF-1α after transfection with MTDH siRNA. Nuclear proteins were extracted, and the level of SREBP1 in the nucleus was detected by immunoblotting. Quantitative analysis of band grayscale values was performed using Image J software. **f** Endothelial cells were treated with Erastin, Erastin combined with SDF-1α, or Erastin combined with SDF-1α after transfection with MTDH siRNA. The subcellular localization of SREBP1 in the endoplasmic reticulum was detected by co-immunofluorescence staining using an endoplasmic reticulum fluorescent probe. **g** Endothelial cells were treated with Erastin, Erastin combined with SDF-1α, or Erastin combined with SDF-1α after transfection with MTDH siRNA. The subcellular localization of SREBP1 in the Golgi apparatus was detected by co-immunofluorescence staining using a Golgi fluorescent probe

Next, MTDH expression in endothelial cells was inhibited by siRNA transfection. Then the cells were treated with Erastin and SDF-1α. Erastin and SDF-1α treatment significantly reduced CCK-8 values in transfected endothelial cells when compared with the negative control group (Fig. 8A). The wound-healing test results showed that the distance between transfected endothelial cells treated with Erastin and SDF-1α significantly broader than that in the negative control group (Fig. 8B). Quantitative analysis of the Matrigel tube formation assay revealed a substantial increase in tube formation by transfected endothelial cells treated with Erastin and SDF-1α exhibited a much wider scratch than the negative control group (Fig. 8C). CM-H2DCFDA analysis indicated that, after transfection, the ROS level in transfected cells was upregulated compared with the negative control group, and this effect was reversed by SDF-1α (Fig. 8D). Immunoblotting showed nuclear SREBP1 level was decreased in transfected cells treated with Erastin and SDF-1α compared with negative control cells (Fig. 8E). SREBP1 localization to the endoplasmic reticulum was decreased, and SREBP1 localization to the Golgi apparatus was increased (Fig. 8F and G).

## Discussion

This study aimed to investigate molecular mechanisms of anti-VEGF drug resistance. In this study, ferroptosis was induced in mouse retina and BMECs by treatment with anti-VEGF drug Ranibizumab treatment. In a recent study, human hepatocellular carcinoma (Hep2) cells with the anti-VEGF drug Bevacizumab, and RNA sequencing was performed. The results showed that Bevacizumab upregulated 37 genes promoting ferroptosis, and downregulated 7 genes that inhibit ferroptosis, indicating that ferroptosis induced by anti-VEGF drugs has important anti-tumor effects (Hou et al. 2024). The tyrosine kinase inhibitor (TKI) Sorafenib also inhibits vascularization by interfering with VEGFR function, but exhibits severe cardiotoxicity. Research has shown that the specific ferroptosis inhibitor ferrostatin-1 and the iron chelator deferoxamine mesylate significantly alleviate Sorafenib-induced cardiac damage, indicating that inhibiting the ferroptosis induced by anti-VEGFR drugs can limit their side effects (Jiang et al. 2022). Therefore, drugs that target and inhibit VEGF/VGEFR signaling can have both therapeutic and toxicity effects. It is generally thought that anti-VEGF drugs resistance mainly occurs through activation of other pathways in parallel with the VEGF pathway and through generating of neutralizing antibodies against the drugs (Mettu et al. 2021); however, our study suggests that lipid metabolism reprogramming induced by SDF-1α after anti-VEGF drug treatment decreases cell sensitivity to ferroptosis, and thus decreases drug resistance. Our findings therefore provide new insight into the mechanisms of anti-VEGF drugs resistance.

Similar to a previous study, we found that the SREBP1/SCD1 pathway decreases cell sensitivity to ferroptosis by regulating lipid peroxidation (Yi et al. 2020). Previous research has mainly focused on changes in SREBP1 expression in disease models (He et al. 2024), but our study explored the regulation of SREBP1 maturation and function, further expanding our knowledge of ferroptosis inhibition. The INSIG1/INSIG2 complex and SEC23A have been reported to mediate the transport of SREBP1 from the ER to Golgi compartments (Xu et al. 2020; Lu et al. 2022), and we found that MTDH also participates in this process. In addition, our study provides new information about SREBP1 maturation.

Our study, however, does have some limitations. Eight-week-old mice correspond to young adult humans. Although the retina of mice at this age is developmentally mature, compared with the middle-aged and elderly populations prone to AMD, they lack age-related baseline pathological features (such as drusen deposition and retinal pigment epithelial (RPE) cell dysfunction) (Fane and Weeraratna 2020). Thus, the model may not accurately reflect the chronic degenerative process that occurs in human AMD. In the laser photocoagulation-induced CNV model used in this study, the choroidal thickness and vascular reactivity in 8-week-old mice may differ from those in middle-aged and elderly mice, potentially causing differences in post-injury repair mechanisms (such as vascular sprouting speed and scar formation ability), which could affect the evaluation of therapeutic responses. While the 8-week-old mouse model provides a convenient tool for CNV research, it has inherent limitations in mimicking the age-related pathology, chronic disease course, and multifactorial pathogenesis of human AMD. Future studies could incorporate middle-aged and elderly mice (such as those aged 12–16 weeks or older) and multimodal induction techniques (such as senescence-accelerated mice subjected to laser injury) to enhance the clinical translation value of the findings.

Another limitation is that we used BMECs instead of retinal endothelial cells (RECs) in the in vitro experiments. When studying AMD pathogenesis, retinal microvascular endothelial cells are preferred for in vitro models. However, laser-induced CNV involves damage to both retinal and choroidal blood vessels. BMECs can be used to independently investigate the universal mechanisms of extraocular vascular lesions, reducing interference from the complex retinal microenvironment. Additionally, BMECs and RECs share the following similarities: both the blood–brain barrier (BBB) and the blood–retinal barrier (BRB) are maintained by endothelial cells, pericytes, specialized basement membrane matrices, and surrounding glial cells (such as astrocytes/Müller cells), and both BMECs and RECs can form vascular barrier with high impedance. When exposed to factors stimulating inflammatory or the formation of vascular lesions (such as VEGF overexpression), the tight junctions between both BMECs and RECs may be disrupted, increasing vascular permeability and simulating BRB leakage in AMD (Gu et al. 2003). Isolating retinal endothelial cells is highly technically difficult, and the mouse or rat cerebral cortex has a large volume high vascular density. In contrast, methods for isolating BMECs (such as enzyme digestion combined with density gradient centrifugation) have been standardized and have higher cell acquisition efficiency. Therefore, we used brain microvascular endothelial cells instead of retinal endothelial cells in this study.

Finally, we explored the role of MTDH in SREBP1 maturation based on phosphoproteomics and protein identification by mass spectrometry, but did not investigate any other differentially modified proteins. We found that SDF-1αdecreases MTDH phosphorylation, but did not investigate the mechanism; this will be the focus of a future study. CXCR4 and CXCR7 are typical SDF-1α receptors that, when bound, activate the downstream PI3K/AKT/ERK pathways (Yang et al. 2023; Fu et al. 2023); however, our study did not involve CXCR4 or CXCR7. Although PI3K/AKT/ERK signaling axis plays a regulatory role in both glycolytic and lipid metabolic pathways (Wang et al. 2019; Fu et al. 2017), previous studies have not shown whether the SDF-1α/CXCR4/CXCR7 pathway regulates glycolysis, lipid metabolism, and amino acid metabolism. Our findings demonstrate pivotal role of metabolic pathways in mediating anti-VEGF resistance, and offering significant clinical implications for improving AMD treatment targets.

## Conclusion

We found that that SDF-1α–mediated promotion of SREBP1 endoplasmic reticulum-to-Golgi transportation and nuclear import inhibits endothelial cells ferroptosis. SREBP1–INSIG1/INSIG2 binding was inhibited by SREBP1–MTDH binding. This study identifies SDF-1α as a critical regulator of ferroptosis resistance in endothelial cells, explaining a fundamental limitation of anti-VEGF drugs treatment, which provides a theoretical basis for clinical solutions to anti-VEGF drug resistance.

## Supplementary Information

Below is the link to the electronic supplementary material.

Supplementary file1 (TIF 1246 KB)

Supplementary file2 (TIF 1376 KB)

Supplementary file3 (XLSX 619 KB)

Supplementary file4 (XLSX 62 KB)

## Data Availability

Data is provided within the manuscript or supplementary information files.

## References

[CR1] Ahmed N, Preisinger C, Wilhelm T, Huber M. TurboID-Based IRE1 interactome reveals participants of the endoplasmic reticulum-associated protein degradation machinery in the human mast cell leukemia cell line HMC-1.2. Cells. 2024;13:747. 10.3390/cells13090747 PMC11082977 38727283

[CR2] Chen J, Zhao R, Wang Y, Xiao H, Lin W, Diao M, He S, Mei P, Liao Y. G protein-coupled estrogen receptor activates PI3K/AKT/mTOR signaling to suppress ferroptosis via SREBP1/SCD1-mediated lipogenesis. Mol Med. 2024;30:28. 38383297 10.1186/s10020-023-00763-x PMC10880371

[CR3] Chang CW, Seibel AJ, Avendano A, Cortes-Medina MG, Song JW. Distinguishing specific CXCL12 isoforms on their angiogenesis and vascular permeability promoting properties. Adv Healthc Mater. 2020;9. 10.1002/adhm.201901399 PMC7033017 31944591

[CR4] Fane M, Weeraratna AT. How the ageing microenvironment influences tumour progression. Nat Rev Cancer. 2020;20:89–106. 31836838 10.1038/s41568-019-0222-9 PMC7377404

[CR5] Flaxel CJ, Adelman RA, Bailey ST, Fawzi A, Lim JI, Vemulakonda GA, Ying GS. Age-related macular degeneration preferred practice pattern(R). Ophthalmology. 2020;127:P1-65. 31757502 10.1016/j.ophtha.2019.09.024

[CR6] Fleckenstein M, Schmitz-Valckenberg S, Chakravarthy U. Age-related macular degeneration: a review. JAMA. 2024;331:147–57. 38193957 10.1001/jama.2023.26074 PMC12935482

[CR7] Fu S, Song X, Hu Y, Zhu Q, Lv X, Tang X, Zhang M. Neotuberostemonine and tuberostemonine ameliorate pulmonary fibrosis through suppressing TGF-beta and SDF-1 secreted by macrophages and fibroblasts via the PI3K-dependent AKT and ERK pathways. Chin J Nat Med. 2023;21:527–39. 37517820 10.1016/S1875-5364(23)60444-3

[CR8] Fu H, He Y, Qi L, Chen L, Luo Y, Chen L, Li Y, Zhang N, Guo H. cPLA2alpha activates PI3K/AKT and inhibits Smad2/3 during epithelial-mesenchymal transition of hepatocellular carcinoma cells. Cancer Lett. 2017;403:260–70. 28649002 10.1016/j.canlet.2017.06.022

[CR9] Gu X, Zhang J, Brann DW, Yu FS. Brain and retinal vascular endothelial cells with extended life span established by ectopic expression of telomerase. Invest Ophthalmol vis Sci. 2003;44:3219–25. 12824274 10.1167/ivos.02-0852

[CR10] He Y, Qi S, Chen L, Zhu J, Liang L, Chen X, Zhang H, Zhuo L, Zhao S, Liu S, Xie T. The roles and mechanisms of SREBP1 in cancer development and drug response. Genes Dis. 2024;11:100987. 38560498 10.1016/j.gendis.2023.04.022 PMC10978545

[CR11] Hou CY, Lv P, Yuan HF, Zhao LN, Wang YF, Zhang HH, Yang G, Zhang XD. Bevacizumab induces ferroptosis and enhances CD8(+) T cell immune activity in liver cancer via modulating HAT1 and increasing IL-9. Acta Pharmacol Sin. 2024;45:1951–63. 38760543 10.1038/s41401-024-01299-4 PMC11335855

[CR12] Jiang X, Stockwell BR, Conrad M. Ferroptosis: mechanisms, biology and role in disease. Nat Rev Mol Cell Biol. 2021;22:266–82. 33495651 10.1038/s41580-020-00324-8 PMC8142022

[CR13] Jiang H, Wang C, Zhang A, Li Y, Li J, Li Z, Yang X, Hou Y. ATF4 protects against sorafenib-induced cardiotoxicity by suppressing ferroptosis. Biomed Pharmacother. 2022;153:113280. 35724508 10.1016/j.biopha.2022.113280

[CR14] Li JH, Li Y, Huang D, Yao M. Role of Stromal Cell-Derived Factor-1 in Endothelial Progenitor Cell-Mediated Vascular Repair and Regeneration, Tissue Eng. Regen Med. 2021;18:747–58. 10.1007/s13770-021-00366-9 PMC8440704 34449064

[CR15] Li Y, Liu X, Zhou T, Kelley MR, Edwards P, Gao H, Qiao X. Inhibition of APE1/Ref-1 redox activity rescues human retinal pigment epithelial cells from oxidative stress and reduces choroidal neovascularization. Redox Biol. 2014;2:485–94. 24624338 10.1016/j.redox.2014.01.023 PMC3949093

[CR16] Liao P, Wang W, Wang W, Kryczek I, Li X, Bian Y, Sell A, Wei S, Grove S, Johnson JK, Kennedy PD, Gijon M, Shah YM, Zou W. CD8(+) T cells and fatty acids orchestrate tumor ferroptosis and immunity via ACSL4. Cancer Cell. 2022;40(365–378):e366. 10.1016/j.ccell.2022.02.003 PMC9007863 35216678

[CR17] Liu A, Liang C, Liu J, Huang Y, Wang M, Wang L. Reactive Oxygen Species horizontal line Responsive Lipid Nanoparticles for Effective RNAi and Corneal Neovascularization Therapy. ACS Appl Mater Interfaces. 2022;14:17022–31. 35380773 10.1021/acsami.1c23412

[CR18] Liu Y, Bao D, She H, Zhang Z, Shao S, Wu Z, Wu Y, Li Q, Wang L, Li T, Liu L. Role of Hippo/ACSL4 axis in ferroptosis-induced pericyte loss and vascular dysfunction in sepsis. Redox Biol. 2024;78:103353. 39566164 10.1016/j.redox.2024.103353 PMC11617880

[CR19] Lu Y, Ma J, Li P, Liu B, Wen X, Yang J. Ilexgenin A restrains CRTC2 in the cytoplasm to prevent SREBP1 maturation via AMP kinase activation in the liver. Br J Pharmacol. 2022;179:958–78. 33434948 10.1111/bph.15369

[CR20] Mettu PS, Allingham MJ, Cousins SW. Incomplete response to Anti-VEGF therapy in neovascular AMD: Exploring disease mechanisms and therapeutic opportunities. Prog Retin Eye Res. 2021;82:100906. 33022379 10.1016/j.preteyeres.2020.100906 PMC10368393

[CR21] Scotti F, Maestroni A, Palini A, Introini U, Setaccioli M, Lorenzi M, Zerbini G. Endothelial progenitor cells and response to ranibizumab in age-related macular degeneration. Retina. 2014;34:1802–10. 24736462 10.1097/IAE.0000000000000147

[CR22] Sun LP, Seemann J, Goldstein JL, Brown MS. Sterol-regulated transport of SREBPs from endoplasmic reticulum to Golgi: Insig renders sorting signal in Scap inaccessible to COPII proteins. Proc Natl Acad Sci U S A. 2007;104:6519–26. 17428919 10.1073/pnas.0700907104 PMC1851663

[CR23] Wang G, Fu XL, Wang JJ, Guan R, Sun Y, Tony To SS. Inhibition of glycolytic metabolism in glioblastoma cells by Pt3glc combinated with PI3K inhibitor via SIRT3-mediated mitochondrial and PI3K/Akt-MAPK pathway. J Cell Physiol. 2019;234:5888–903. 29336479 10.1002/jcp.26474

[CR24] Xu D, Wang Z, Xia Y, Shao F, Xia W, Wei Y, Li X, Qian X, Lee JH, Du L, Zheng Y, Lv G, Leu JS, Wang H, Xing D, Liang T, Hung MC, Lu Z. The gluconeogenic enzyme PCK1 phosphorylates INSIG1/2 for lipogenesis. Nature. 2020;580:530–5. 32322062 10.1038/s41586-020-2183-2

[CR25] Yang Y, Li J, Lei W, Wang H, Ni Y, Liu Y, Yan H, Tian Y, Wang Z, Yang Z, Yang S, Yang Y, Wang Q. CXCL12-CXCR4/CXCR7 Axis in Cancer: from Mechanisms to Clinical Applications. Int J Biol Sci. 2023;19:3341–59. 37497001 10.7150/ijbs.82317 PMC10367567

[CR26] Yi J, Zhu J, Wu J, Thompson CB, Jiang X. Oncogenic activation of PI3K-AKT-mTOR signaling suppresses ferroptosis via SREBP-mediated lipogenesis. Proc Natl Acad Sci USA. 2020;117:31189–97. 33229547 10.1073/pnas.2017152117 PMC7733797

[CR27] Zhang C, Liu X, Jin S, Chen Y, Guo R. Ferroptosis in cancer therapy: a novel approach to reversing drug resistance. Mol Cancer. 2022;21:47. 35151318 10.1186/s12943-022-01530-y PMC8840702

[CR28] Zheng D, Liu J, Piao H, Zhu Z, Wei R, Liu K. ROS-triggered endothelial cell death mechanisms: Focus on pyroptosis, parthanatos, and ferroptosis. Front Immunol. 2022;13:1039241. 36389728 10.3389/fimmu.2022.1039241 PMC9663996

[CR29] Zhong C, Wang J, Li B, Xiang H, Ultsch M, Coons M, Wong T, Chiang NY, Clark S, Clark R, Quintana L, Gribling P, Suto E, Barck K, Corpuz R, Yao J, Takkar R, Lee WP, Damico-Beyer LA, Carano RD, Adams C, Kelley RF, Wang W, Ferrara N. Development and preclinical characterization of a humanized antibody targeting CXCL12. Clin Cancer Res. 2013;19:4433–45. 23812669 10.1158/1078-0432.CCR-13-0943

[CR30] Zou Y, Palte MJ, Deik AA, Li H, Eaton JK, Wang W, Tseng YY, Deasy R, Kost-Alimova M, Dancik V, Leshchiner ES, Viswanathan VS, Signoretti S, Choueiri TK, Boehm JS, Wagner BK, Doench JG, Clish CB, Clemons PA, Schreiber SL. A GPX4-dependent cancer cell state underlies the clear-cell morphology and confers sensitivity to ferroptosis. Nat Commun. 2019;10:1617. 30962421 10.1038/s41467-019-09277-9 PMC6453886

