# Supplementary figures and images for: The SDF-1α/MTDH axis inhibits ferroptosis and promotes the formation of anti-VEGF-resistant choroidal neovascularization by facilitating the nuclear translocation of SREBP1
Source: Cell Biol Toxicol. 2025 Jul 17;41(1):118. doi: 10.1007/s10565-025-10066-y (PMC12267381; doi:10.1007/s10565-025-10066-y)

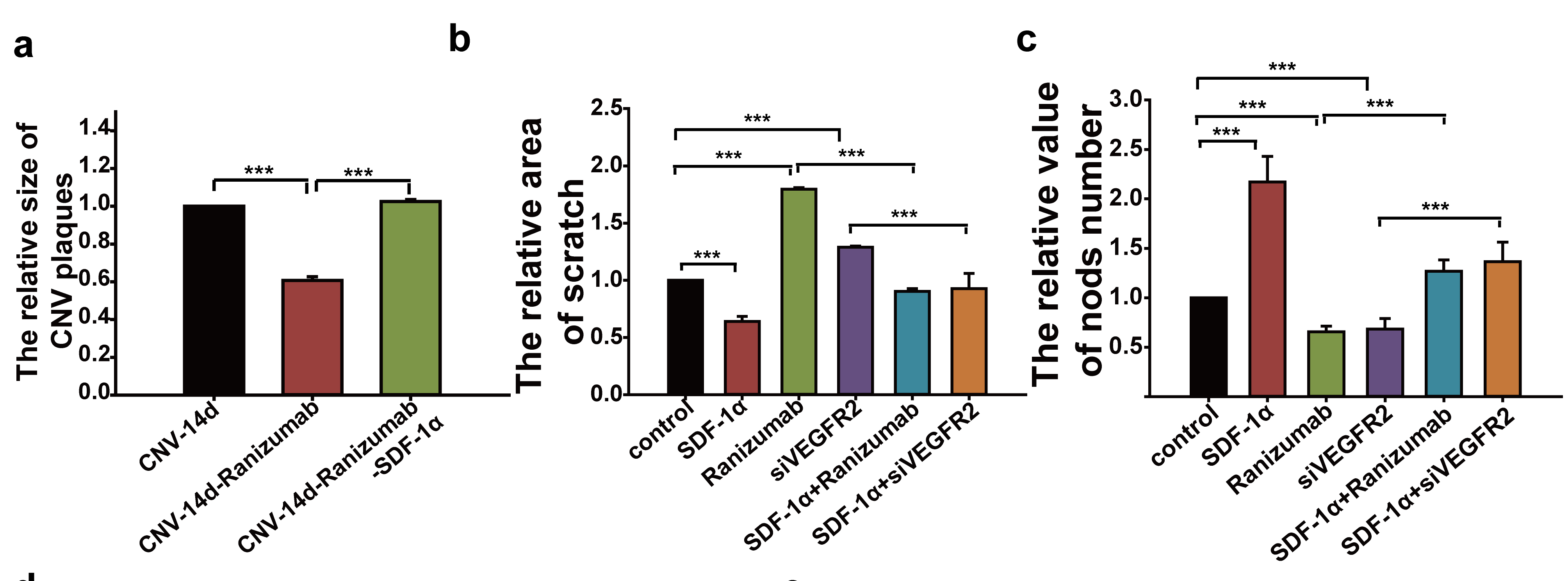

Supplement: Supplementary file 1 — Supplementary file1 (TIF 1246 KB) [file 10565_2025_10066_MOESM1_ESM.tif]

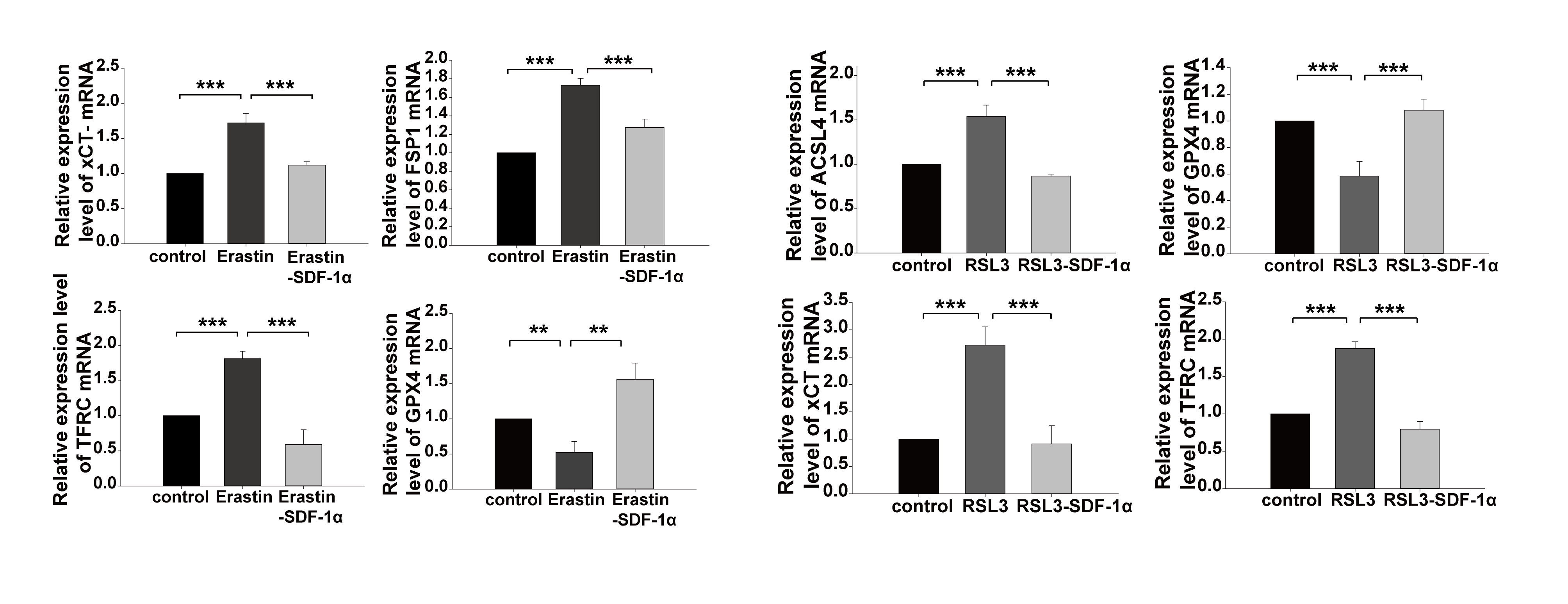

Supplement: Supplementary file 2 — Supplementary file2 (TIF 1376 KB) [file 10565_2025_10066_MOESM2_ESM.tif]
